# A comparative analysis of graph neural networks and classical machine learning for carbapenem-resistance prediction and genomic interpretation in *Acinetobacter baumannii*

**DOI:** 10.3389/fbinf.2026.1885813

**Published:** 2026-09-28

**Authors:** Irfan Sharif Shaik, Morteza Sarmadi

**Affiliations:** 1 Hercules High School, Hercules, CA, United States; 2 Massachusetts Institute of Technology Alumni Association, Cambridge, MA, United States

**Keywords:** *Acinetobacter baumannii*, antimicrobial resistance (AMR), bacterial genomics, carbapenem resistance, graph neural networks (GNNs), model interpretability, pangenome analysis, random forest

## Abstract

**Introduction:**

Carbapenem-resistant *Acinetobacter baumannii* is a hospital-associated pathogen linked to high mortality and limited treatment options. Although many resistance determinants have been identified, how they organize into coordinated systems remains unclear.

**Methods:**

Using a final binary modeling dataset of 2,448 genomes, we benchmarked machine-learning and graph-based models and developed a framework that pairs accurate prediction with systems-level genomic interpretation.

**Results:**

Across five random seeds and 10-fold stratified cross-validation, a Random Forest classifier achieved the strongest internal performance using a bag-of-genes representation, with an accuracy of 0.9328 ± 0.0175 and ROC-AUC of 0.9650 ± 0.0138. Random Forest also generalized best in an independent BV-BRC Asia validation cohort of 2,500 genomes, achieving an accuracy of 0.7728 and ROC-AUC of 0.8392. In parallel, a Graph Convolutional Network trained on gene-adjacency graphs enabled interpretation through *post hoc* explainability, prioritizing genes consistent with a candidate three-layer resistance architecture: (i) canonical rapid-response resistance genes, (ii) a cell-surface “Sugar Shield” centered on GDP/UDP-epimerase, and (iii) a metabolic “Command Center” involving regulators such as DnaA and quinone oxidoreductase 1. Explanation-stability analysis identified a reproducible consensus subset while supporting cautious interpretation of individual rankings. Complementary computational analyses, including protein–protein interaction enrichment, Gene Ontology enrichment, structural modeling, and molecular docking, supported the biological coherence and structural plausibility of the proposed architecture.

**Discussion:**

Together, these findings suggest that carbapenem resistance may reflect a coordinated, multi-layer defense system and nominate GDP/UDP-epimerase and quinone oxidoreductase 1 as candidates for future experimental investigation.

## 1 Introduction

Carbapenem-resistant *Acinetobacter baumannii* (CRAB) is a hospital-associated pathogen linked to high mortality with limited treatment options (Bartal et al., 2022; World Health Organization, 2024; Boutzoukas and Doi, 2025). Carbapenems are often used as last-line antibiotics, and CRAB infections are difficult to treat once resistance emerges. Carbapenem resistance in *Acinetobacter baumannii* is most commonly associated with acquired carbapenem-hydrolyzing class D β-lactamases, particularly OXA-23, although altered permeability, efflux systems, insertion-sequence-mediated expression, and envelope-regulatory responses can also contribute. These mechanisms operate within broader cellular and genomic contexts rather than as fully independent determinants (Marchand et al., 2004; Geisinger et al., 2018; Hamidian and Nigro, 2019). In this study, “systems-level” refers to the coordinated interactions among multiple genes and pathways in response to antibiotic stress. Rather than only listing which resistance genes are present, we asked whether resistance follows an organized pattern across functional layers (e.g., drug inactivation, surface remodeling, and metabolic adaptation). Understanding this organization can improve genomic prediction of resistance and highlight therapeutic vulnerabilities that may be missed when genes are analyzed individually.

Phenotypic antimicrobial susceptibility testing remains the clinical reference for determining resistance, while targeted molecular assays can rapidly detect known resistance determinants. Genome-wide computational approaches broaden this strategy by learning resistance-associated patterns from pan-genome, k-mer, and other sequence-derived representations. Previous studies have demonstrated that Logistic Regression, Support Vector Machines, Random Forests, and neural networks can predict antimicrobial-resistance phenotypes from genome-scale features across bacterial species (Moradigaravand et al., 2018; Aytan-Aktug et al., 2020). In *A. baumannii* specifically, machine-learning and neural-network approaches have been applied to whole-genome sequencing, metagenomic sequencing, and gene-expression data for antimicrobial-susceptibility prediction (Hu et al., 2023; Gao et al., 2024; Jia et al., 2024). Broader benchmarking studies have also evaluated the performance and limitations of computational AMR prediction across bacterial species and genome-derived representations (Hu et al., 2024).

Graph neural networks provide an additional modeling strategy by explicitly aggregating information across graph-connected features. Foundational architectures include Graph Convolutional Networks, GraphSAGE, and Graph Attention Networks (Kipf and Welling, 2017; Hamilton et al., 2017; Veličković et al., 2018). These developments have also been reviewed across bioinformatics and broader graph-learning applications (Zhang et al., 2021; Khemani et al., 2024). More recently, multi-representation GNN frameworks have been applied directly to genomic antimicrobial-resistance prediction, reflecting growing interest in relational genome representations for this task (Nguyen et al., 2026). However, GNNs are not inherently interpretable. Their predictions still require *post hoc* explanation, and explanation quality, fidelity, and stability must be evaluated explicitly (Ying et al., 2019; Agarwal et al., 2023).

Existing approaches therefore involve complementary trade-offs rather than a single universally superior representation. Targeted molecular methods are transparent and rapid but are primarily restricted to known determinants. Vectorized genome-wide models can achieve strong predictive performance and can capture nonlinear feature combinations, but they do not explicitly represent genomic adjacency or other predefined biological relationships. Graph models can encode relational structure directly, but they introduce additional modeling complexity and still require *post hoc* interpretation and stability assessment. Moreover, strong internal cross-validation performance does not necessarily guarantee transfer across bacterial populations, geographic regions, or data-generation settings, making independent external validation and population-aware evaluation important for genomic AMR prediction (Verschuuren et al., 2022; Yu et al., 2025).

Here, we combine feature-based and graph-based learning to address both prediction and biological interpretation. Using a final binary modeling dataset of 2,448 genomes, we benchmarked Prokka-derived gene-presence models and graph-based genome representations for carbapenem-resistance prediction. The benchmark included classical and feature-based models, graph neural networks, an exploratory random-walk visit-frequency baseline, and an exploratory bag-of-genes Transformer encoder. We further evaluated external generalization using an independent BV-BRC Asia cohort and assessed the stability of GCN explanations across multiple random seeds. This design allowed us to compare predictive performance, external transferability, and explanation-derived biological signals within a single framework. The Random Forest model provided the strongest overall predictive and external-validation performance, whereas the GCN was used as a complementary graph-based model for mechanism-oriented explanation and candidate biological hypothesis generation. An overview of the study workflow is shown in Figure 1.

**FIGURE 1:**
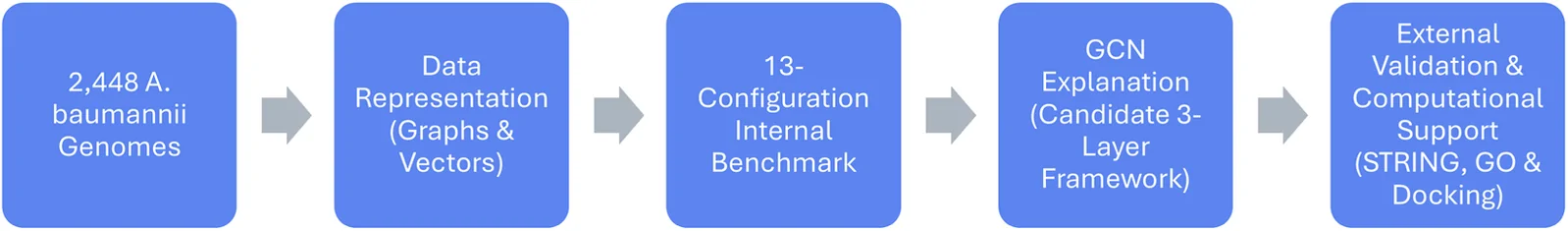
Overview of the study workflow. The final internal binary cohort of 2,448 *Acinetobacter baumannii* genomes was represented using graph-based and vector-based encodings and evaluated through an expanded 13-configuration internal benchmark. The gene-based GCN was subsequently used for explanation and stability analyses to prioritize genes consistent with a candidate three-layer resistance framework. Independent external validation and complementary STRING protein-association, Gene Ontology enrichment, structural-modeling, and molecular-docking analyses were used to evaluate predictive transferability and provide computational support for selected candidates.

Explainability analysis of the GCN prioritized genes consistent with a candidate Three-Layer Resistance Architecture composed of: (1) canonical resistance and efflux-related genes, (2) a cell-surface biosynthesis layer centered on GDP/UDP-epimerase, and (3) a metabolic regulation layer involving candidates such as DnaA and quinone oxidoreductase 1. We evaluated the coherence of this architecture through cross-model comparison, explanation-stability analysis, susceptible-versus-resistant explanation comparison, STRING protein-association enrichment, Gene Ontology enrichment, and molecular docking. Overall, this work advances both prediction and biological interpretation by providing: (i) an expanded benchmark for carbapenem-resistance prediction, (ii) independent external validation on a geographically distinct cohort, (iii) seed-stability analysis for GNNExplainer outputs, and (iv) a graph-explanation framework for prioritizing candidate resistance-associated systems for future experimental study.

## 2 Methods

### 2.1 Data acquisition and phenotype curation

Genomic and antimicrobial susceptibility metadata for *Acinetobacter baumannii* were obtained from the Bacterial and Viral Bioinformatics Resource Center (BV-BRC, formerly PATRIC) (Olson et al., 2023). The initial dataset contained 2,569 isolates associated with 22,983 antibiotic-susceptibility testing (AST) records. To define a carbapenem-resistance prediction task, AST records associated with carbapenem antibiotics, including imipenem, meropenem, and doripenem, were retained and standardized into resistant, susceptible, intermediate, or non-informative phenotype categories.

After phenotype curation, the curated dataset contained 2,450 genomes. Because this study was designed as a binary resistant-versus-susceptible classification task, the two genomes labeled as intermediate were excluded from model training and evaluation. The final internal binary modeling cohort therefore contained 2,448 genomes, including 1,815 susceptible genomes and 633 resistant genomes. Susceptible genomes were encoded as 0 and resistant genomes were encoded as 1. Genome identifiers were standardized across phenotype metadata, Prokka annotation files, bag-of-genes matrices, and graph files to ensure consistent sample alignment.

### 2.2 Prokka annotation and genome feature extraction

All genomes were annotated with Prokka (Seemann, 2014). Prokka general feature format (GFF) files were used as the primary source for gene/product-level feature extraction. Coding sequence (CDS) entries were parsed from each GFF file, and gene/product names were extracted from the annotation attributes.

For bag-of-genes feature extraction, the primary feature name was taken from the “product” field when available. If the “product” field was missing, fallback fields were used in the following order: “gene”, “Name”, “locus_tag”, and “ID”. Empty, missing, or uninformative feature names were excluded. For each genome, the set of observed Prokka-derived gene/product annotations was converted into a binary vector indicating feature presence or absence.

This procedure produced the final internal bag-of-genes matrix containing 2,448 genomes and 5,606 Prokka-derived gene/product features. This matrix was used for Logistic Regression, Random Forest, Linear SVM, XGBoost, and the exploratory bag-of-genes Transformer encoder.

### 2.3 Genome representations

Two complementary genome representations were constructed: graph-based representations for graph neural network analysis and vector-based representations for classical feature-based machine learning. The four genome-representation strategies are illustrated schematically in Figure 2.

**FIGURE 2:**
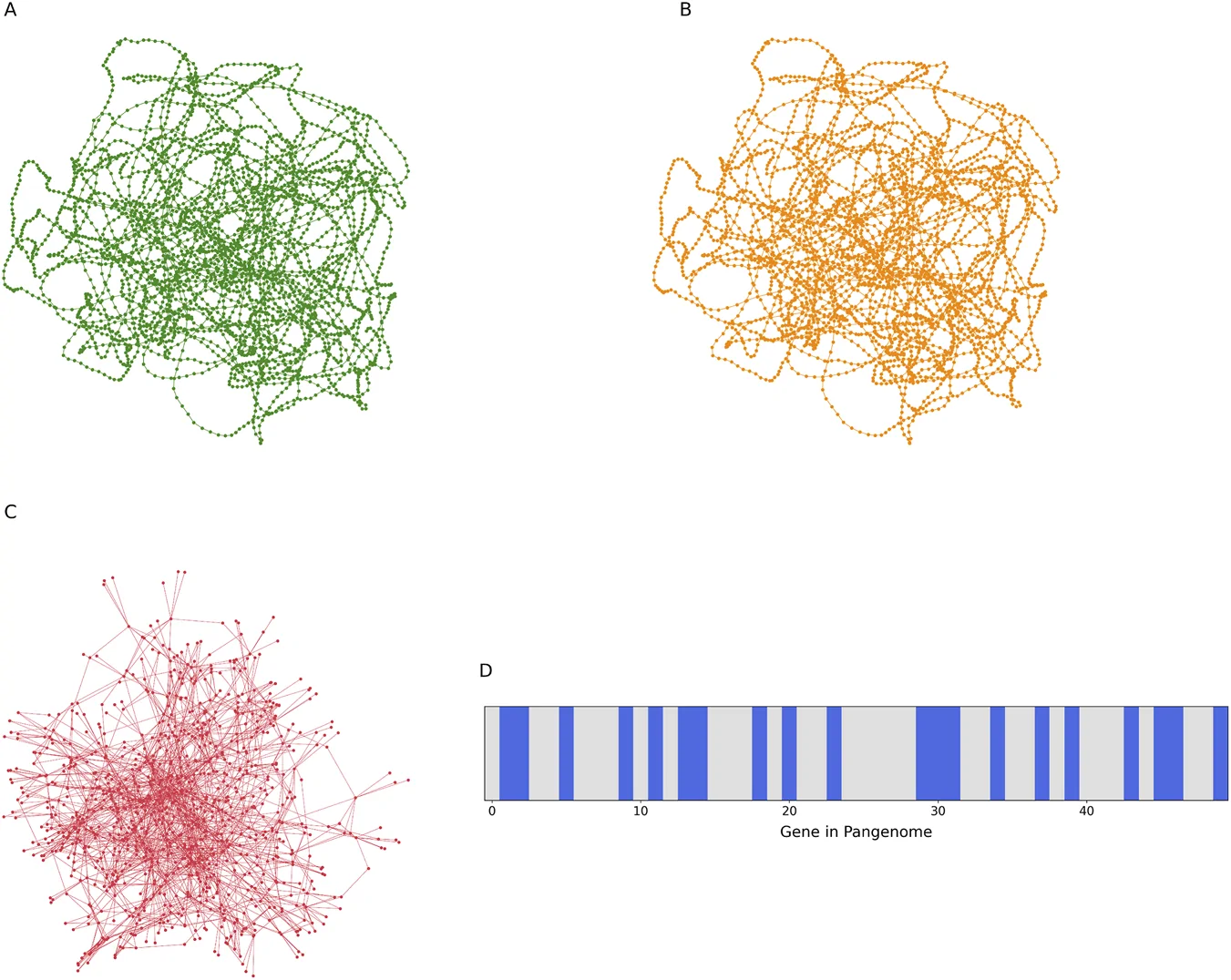
Schematic representation of the genome encodings used for model training. **(A)** Gene-based graph in which nodes represent Prokka-derived gene/product features and edges connect genomically adjacent coding sequences. **(B)** Hybrid graph retaining the gene-adjacency topology while enriching each node with a feature-hashed 6-mer profile. **(C)** Subsampled k-mer graph in which nodes represent contiguous 6-mers and edges connect sequential k-mers. **(D)** Illustrative bag-of-genes vector in which blue and gray positions indicate the presence and absence, respectively, of features from the global pangenome vocabulary. The bag-of-genes representation was used by the feature-based classifiers and Transformer encoder.

#### 2.3.1 Graph-based encodings

Three graph-based encodings were generated from the Prokka annotations:

1. Gene-based graph: nodes correspond to Prokka-derived gene/product annotations, and edges connect adjacent loci.
2. k-mer graph: nodes represent contiguous 6-mers, and edges connect sequential k-mers.
3. Hybrid graph: the gene-graph topology is retained, but each gene node is enriched with a feature-hashed k-mer profile.

For the hybrid representation, overlapping 6-mers were extracted from each gene sequence, with reverse-complement handling for genes on the negative strand. Each 6-mer was hashed with MD5 and mapped into one of 512 bins using modulo indexing. Bin counts were accumulated as raw counts, with hash collisions summed implicitly.

#### 2.3.2 Vector-based bag-of-genes encoding

For classical machine-learning algorithms, each genome was represented as a binary bag-of-genes vector. Each column represented one Prokka-derived gene/product feature from the global internal vocabulary, and each value indicated whether that feature was present or absent in a given genome. This representation enabled direct comparison among linear, ensemble, margin-based, gradient-boosted, and Transformer-style classifiers using the same 5,606-feature input space.

### 2.4 Gene-adjacency graph construction

For the gene-based graph representation, each *A. baumannii* genome was represented as a gene-level graph derived from Prokka-annotated GFF files.

#### 2.4.1 Graph construction steps

1. Global vocabulary construction:

A global pangenome vocabulary was constructed by scanning Prokka-derived gene/product annotations across the internal cohort and assigning a numeric index to each unique gene/product feature.

2. Genome-level node extraction:

For each genome, the ordered list of CDS features was parsed from the corresponding GFF file. Valid gene/product annotations were mapped to their global vocabulary indices. The set of unique indices for that genome formed the graph node list.

3. Adjacency-edge construction:

Consecutive CDS entries were connected according to their order in the genome annotation. For each adjacent pair in the ordered CDS list, an edge was added from gene *i* to gene *i* + 1. Edges therefore represented local genomic adjacency between annotated coding sequences.

4. PyTorch Geometric conversion:

The graph edges were converted into a PyTorch Geometric edge_index tensor of shape [2, E], where E is the number of graph edges. This procedure produced a directed, unweighted graph for each genome. Genomes with fewer than two annotated genes were excluded to avoid degenerate graphs.

The final binary gene-graph dataset used for the revised GCN analyses contained 2,448 graphs. The graph dataset had a mean of approximately 1,719.8 nodes per graph and 3,823.2 edges per graph. Additional internal-cohort, feature-matrix, graph-structure, and external-cohort summary statistics are provided in Supplementary Table S3.

### 2.5 Internal model benchmarking

Internal model performance was evaluated using stratified cross-validation so that resistant and susceptible class proportions were preserved across held-out folds. The benchmark compared graph-based models trained on genome graph representations with feature-based models trained on the Prokka-derived bag-of-genes matrix.

#### 2.5.1 Graph models

The graph-based benchmark included three graph neural network architectures: Graph Convolutional Network (GCN) (Kipf and Welling, 2017), Graph Attention Network (GAT) (Veličković et al., 2018), and GraphSAGE (Hamilton et al., 2017). Each architecture was evaluated on three graph representations: gene-based graphs, k-mer graphs, and hybrid gene/k-mer graphs, yielding nine graph-model configurations. These models were used to assess whether graph architecture and genome representation influenced carbapenem-resistance prediction.

#### 2.5.2 Feature-based and exploratory models

The feature-based benchmark included Logistic Regression, Random Forest, Linear SVM, and XGBoost trained on the 5,606-feature Prokka bag-of-genes matrix. In addition, two exploratory benchmark models were evaluated: a Random Walk visit-frequency baseline using gene-adjacency graph traversal features, and a bag-of-genes Transformer encoder using tokenized Prokka gene-presence features. Together, these models allowed comparison across linear, ensemble, margin-based, gradient-boosted, graph neural network, graph-traversal, and Transformer-style classifiers.

#### 2.5.3 Model settings

Logistic Regression was trained on the Prokka bag-of-genes matrix using L2 regularization, C = 1.0, the LBFGS solver, a maximum of 1,000 iterations, and no class weighting. Random Forest was trained using 100 trees, Gini impurity splitting, no maximum-depth constraint, square-root feature sampling, bootstrap sampling, and balanced class weights (Breiman, 2001). Linear SVM was implemented using LinearSVC with C = 1.0, class-weight balancing, and a maximum of 5,000 iterations (Cortes and Vapnik, 1995). Because LinearSVC does not directly provide calibrated probabilities, sigmoid calibration with three-fold internal calibration was used to obtain probability estimates for ROC-AUC and threshold-based metrics. XGBoost was implemented as a gradient-boosted tree classifier using a binary logistic objective, 300 estimators, maximum depth 4, learning rate 0.05, subsample 0.9, column subsample 0.9, histogram-based tree construction, log-loss evaluation, and positive-class weighting based on the susceptible-to-resistant class ratio in the training data (Chen and Guestrin, 2016).

#### 2.5.4 Cross-validation and metrics

Unless otherwise specified, repeated-seed analyses used random seeds 42, 123, 456, 789, and 1,000, with 10-fold stratified cross-validation performed for each seed, yielding 50 total held-out test folds. Model performance was evaluated using held-out predictions only. Metrics included accuracy, ROC-AUC, precision, recall/sensitivity, specificity, F1-score, and confusion-matrix counts. Specificity was calculated as TN/(TN + FP). For repeated-seed analyses, metrics are reported as mean ± standard deviation across folds and seeds, and aggregate confusion matrices were computed by summing held-out predictions across all folds and seeds. The seed-42 comparison presented in Figure 3 reports mean accuracy across 10 stratified folds for all 13 internal model configurations. The detailed benchmark presented in Table 1 reports repeated five-seed evaluations for Random Forest, Logistic Regression, Linear SVM, XGBoost, the gene-based GCN, the exploratory bag-of-genes Transformer encoder, and the Random Walk visit-frequency baseline. Each repeated-seed evaluation used random seeds 42, 123, 456, 789, and 1,000 with 10 stratified folds per seed, yielding 50 held-out fold evaluations per model. Model architectures, hyperparameters, class-weighting procedures, and evaluation settings for the 13-configuration seed-42 comparison and the seven-model repeated five-seed benchmark are summarized in Supplementary Table S4.

**FIGURE 3:**
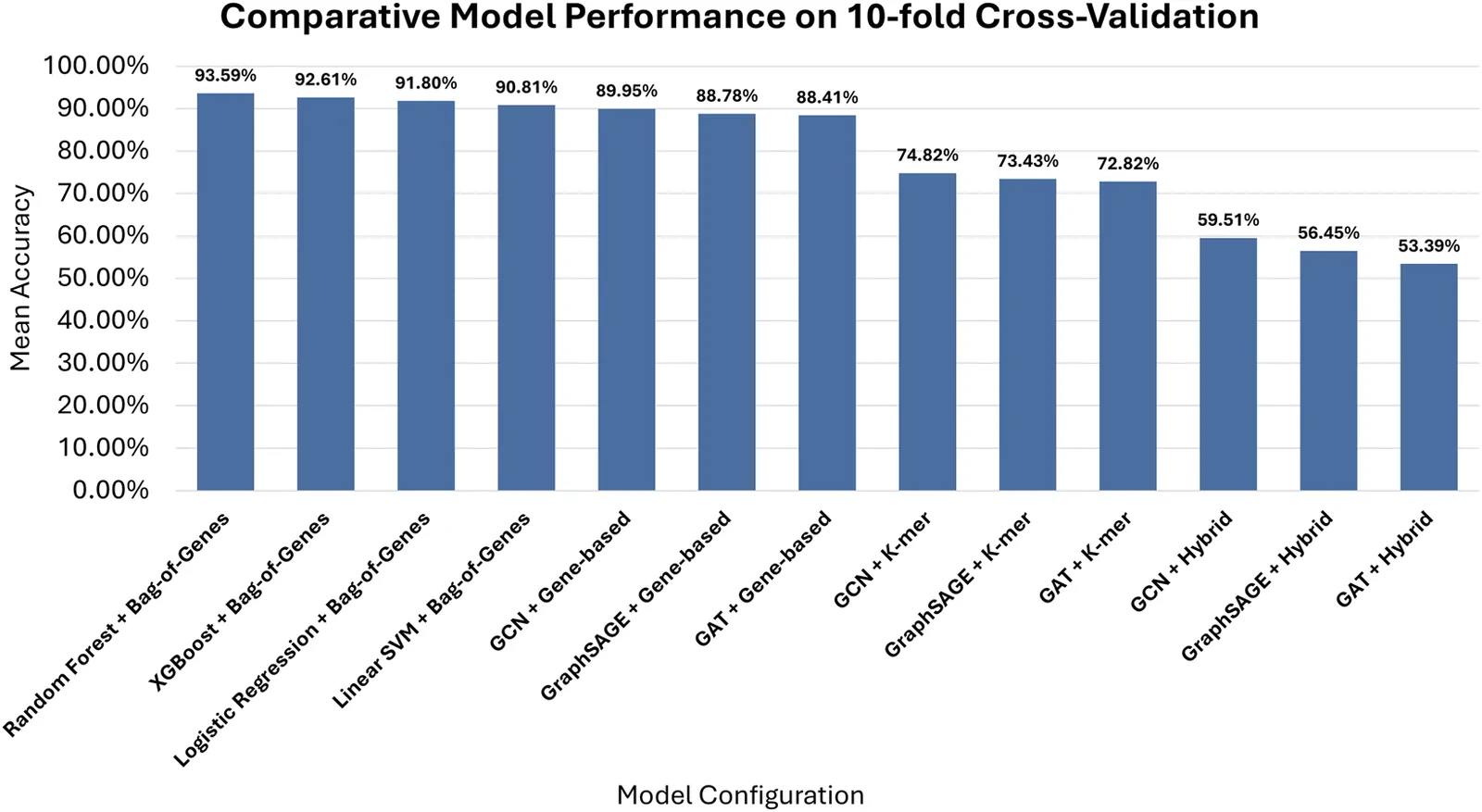
Comparative model performance across 13 internal model configurations. Mean held-out accuracy is shown for 13 model configurations evaluated using stratified 10-fold cross-validation with random seed 42 on the final binary cohort of 2,448 *Acinetobacter baumannii* genomes. The comparison includes four classifiers trained on Prokka-derived bag-of-genes features—Random Forest, XGBoost, Logistic Regression, and Linear SVM—and nine graph-neural-network configurations consisting of GCN, GraphSAGE, and GAT architectures evaluated with gene-based, k-mer-based, and hybrid graph representations. Random Forest achieved the highest mean accuracy at 93.59%, followed by XGBoost at 92.61%, Logistic Regression at 91.80%, and Linear SVM at 90.81%. Among the graph-based configurations, the gene-based GCN achieved the highest mean accuracy at 89.95%. Bars are arranged in descending order of mean accuracy. This seed-42 accuracy-only comparison is distinct from the repeated five-seed benchmark reported in Table 1.

**TABLE 1:** Detailed internal benchmark of classical, graph-based, random-walk, and Transformer-style models for carbapenem-resistance prediction.

Model	Representation	Evaluation	Accuracy	ROC-AUC	Recall/Sensitivity	F1
Random forest	Prokka bag-of-genes	5 seeds × 10-fold stratified CV	0.9328 ± 0.0175	0.9650 ± 0.0138	0.8284 ± 0.0525	0.8639 ± 0.0367
XGBoost	Prokka bag-of-genes	5 seeds × 10-fold stratified CV	0.9297 ± 0.0199	0.9646 ± 0.0154	0.8813 ± 0.0516	0.8664 ± 0.0376
Linear SVM	Prokka bag-of-genes	5 seeds × 10-fold stratified CV	0.9032 ± 0.0179	0.9415 ± 0.0204	0.7044 ± 0.0587	0.7891 ± 0.0427
Logistic regression	Prokka bag-of-genes	5 seeds × 10-fold stratified CV	0.9175 ± 0.0206	0.9541 ± 0.0187	0.8061 ± 0.0598	0.8343 ± 0.0429
Gene-based GCN	Gene-adjacency graph	5 seeds × 10-fold stratified CV	0.8963 ± 0.0190	0.9460 ± 0.0196	0.7567 ± 0.1024	0.7884 ± 0.0460
Bag-of-genes transformer encoder	Tokenized Prokka bag-of-genes	5 seeds × 10-fold stratified CV	0.8823 ± 0.0260	0.9380 ± 0.0182	0.8083 ± 0.0630	0.7813 ± 0.0388
Random walk visit-frequency + logistic regression	Gene-graph visit-frequency features	5 seeds × 10-fold stratified CV	0.5375 ± 0.0337	0.5581 ± 0.0410	0.5431 ± 0.0696	0.3774 ± 0.0401

Seven principal models were evaluated using stratified 10-fold cross-validation conducted independently for random seeds 42, 123, 456, 789, and 1,000, yielding 50 held-out fold evaluations per model. Values are reported as mean ± standard deviation across the 50 held-out folds. Resistance was treated as the positive class. The GCN result corresponds to the gene-based GCN, the Transformer result corresponds to the exploratory encoder trained on tokenized Prokka-derived bag-of-genes features, and the Random Walk result corresponds to the exploratory graph-level visit-frequency baseline classified using Logistic Regression. Figure 3 separately presents the seed-42, mean-accuracy comparison across all 13 internal model configurations. Supplementary Table S5 contains two worksheets: the complete seed-42, accuracy comparison across all 13 configurations and the full repeated five-seed metric set for the seven principal models reported here, including accuracy, ROC-AUC, precision, recall/sensitivity, specificity, F1-score, and aggregate held-out confusion-matrix totals.

### 2.6 GCN architecture and training protocol

The final gene-based GCN used the Prokka-derived gene-adjacency graph representation.

#### 2.6.1 GCN architecture

Each graph used an embedding layer that mapped integer gene/product vocabulary indices to 128-dimensional learned embeddings. The embedded node features were passed through two GCNConv layers, each followed by ReLU activation. Graph-level representations were computed using global mean pooling, which aggregates node embeddings into a single graph-level vector by taking the element-wise mean across nodes. A final linear layer produced a single logit for binary resistant-versus-susceptible classification.

The forward pass consisted of:

1. Embedding node indices using a learnable 128-dimensional embedding matrix.
2. Applying the first graph convolutional layer with ReLU activation.
3. Applying the second graph convolutional layer with ReLU activation.
4. Aggregating node embeddings using global mean pooling.
5. Passing the pooled graph representation through a linear output layer to produce a binary logit.

#### 2.6.2 GCN training protocol

The selected GCN configuration used:

1. Embedding dimension = 128
2. Hidden channels = 128
3. Learning rate = 0.001
4. Batch size = 16
5. Training epochs = 20
6. Optimizer = Adam (Kingma and Ba, 2015)
7. Loss function = BCEWithLogitsLoss

Predicted probabilities were obtained by applying the sigmoid function to the output logit. Binary predictions were generated using a probability threshold of 0.5.

The final GCN robustness analysis was repeated across random seeds 42, 123, 456, 789, and 1,000, with 10 stratified folds per seed. Python, NumPy, PyTorch, and CUDA random seeds were fixed where applicable, and deterministic PyTorch backend settings were used when available.

### 2.7 GCN hyperparameter sensitivity analysis

To evaluate whether the selected GCN configuration was stable, a focused hyperparameter sensitivity analysis was performed across learning rate and hidden-channel settings. This analysis was designed to test key training choices rather than exhaustively search the full GCN hyperparameter space.

The tested configurations were:

1. Learning rate 0.01, hidden channels 128, epochs 20
2. Learning rate 0.001, hidden channels 128, epochs 20
3. Learning rate 0.0001, hidden channels 128, epochs 20
4. Learning rate 0.001, hidden channels 64, epochs 20
5. Learning rate 0.001, hidden channels 256, epochs 20

All configurations used embedding dimension 128, batch size 16, Adam optimization, BCEWithLogitsLoss, and 10-fold stratified cross-validation.

### 2.8 External validation on BV-BRC Asia genomes

To evaluate generalization beyond the internal cohort, an independent external validation cohort was assembled from BV-BRC *A. baumannii* genomes assigned to Asia. Genome identifiers overlapping with the internal cohort were excluded, and only genomes with an unambiguous resistant or susceptible carbapenem phenotype were eligible. Metadata-based assembly plausibility checks used an expected genome-size range of 3,000,000–5,000,000 bp and an expected coding-sequence-count range of 1,000–7,000 CDS. These ranges were recorded as quality-control indicators for the candidate pool rather than treated as absolute exclusion criteria.

External genome FASTA files were downloaded and checked for successful retrieval and usable sequence content before reannotation with Prokka using the same annotation-based feature-extraction strategy as the internal cohort. A target of 2,500 successfully annotated genomes was used so that the external cohort would exceed the 2,448-genome internal cohort. A Prokka annotation was considered complete only when the output contained both a completion marker and a non-empty GFF file larger than 1,000 bytes. The final external validation matrix therefore contained 2,500 genomes, including 1,352 susceptible and 1,148 resistant genomes. The external matrix was aligned to the same 5,606-feature internal bag-of-genes vocabulary, with features absent from an external genome encoded as 0.

#### 2.8.1 External validation design

External validation was performed by training models on the internal 2,448-genome cohort and evaluating them on the independent 2,500-genome external cohort. Thresholds were selected using internal cross-validation only and then applied unchanged to the external cohort, preventing threshold selection from using external test labels.

For each model, internal out-of-fold prediction scores were generated using 10-fold stratified cross-validation. Candidate thresholds were evaluated on the internal out-of-fold scores, and the threshold maximizing internal balanced accuracy was selected. The final model was then trained on the full internal cohort, applied to the external cohort, and evaluated using accuracy, balanced accuracy, ROC-AUC, precision, recall/sensitivity, specificity, F1-score, and confusion-matrix counts.

#### 2.8.2 External-validation model settings

The external-validation models used the following settings:

1. Logistic Regression: L2 penalty, C = 1.0, liblinear solver, class-weight balancing, maximum 5,000 iterations.
2. Random Forest: 500 trees, no maximum depth, minimum split size 2, minimum leaf size 1, balanced-subsample class weighting.
3. Linear SVM: C = 1.0, class-weight balancing, maximum 50,000 iterations, with decision-function scores used for thresholding.
4. XGBoost: 500 estimators, maximum depth 4, learning rate 0.05, subsample 0.8, column subsample 0.8, binary logistic objective, log-loss evaluation, and positive-class weighting based on the internal training class ratio.

### 2.9 Random walk visit-frequency baseline

To evaluate whether simple graph traversal statistics captured resistance-associated signal, we implemented an exploratory Random Walk visit-frequency baseline using the gene-adjacency graphs.

For each genome graph:

1. Random walks were simulated over the gene-adjacency graph structure.
2. The analysis used 100 walks per graph.
3. Each walk had length 15.
4. Visited gene/node identifiers were counted.
5. Counts were normalized by the total number of visits to create graph-level visit-frequency features.

The resulting visit-frequency vectors were used as input to a balanced Logistic Regression classifier with L2 penalty, C = 1.0, the liblinear solver, and maximum 2,000 iterations. The Random Walk baseline was evaluated across seeds 42, 123, 456, 789, and 1,000 with 10-fold stratified cross-validation per seed.

This baseline should be interpreted specifically as a graph-level random-walk visit-frequency model. It was not a DeepWalk or node2vec embedding model and did not learn unsupervised node embeddings of the type introduced by Perozzi et al. (2014) or Grover and Leskovec (2016).

### 2.10 Bag-of-genes transformer encoder

To evaluate a Transformer-style classifier within the same feature space as the classical machine-learning models, we implemented an exploratory bag-of-genes Transformer encoder using the 5,606 Prokka-derived gene-presence features and the self-attention architecture introduced by Vaswani et al. (2017). This model was designed for direct comparison with the Logistic Regression, Random Forest, Linear SVM, and XGBoost benchmarks because it used the same curated binary gene-feature matrix. It should not be interpreted as a raw whole-genome sequence Transformer.

#### 2.10.1 Input tokenization

Each binary bag-of-genes vector was padded from 5,606 features to 5,632 features and divided into 88 tokens of 64 features each. Each token was projected into a 64-dimensional embedding space. A learnable classification token and positional embeddings were added before Transformer encoding.

#### 2.10.2 Transformer architecture and training

The Transformer encoder used:

1. Model dimension = 64
2. Attention heads = 4
3. Encoder layers = 2
4. Feed-forward dimension = 128
5. Dropout = 0.10
6. Activation = GELU
7. Batch size = 64
8. Epochs = 20
9. Optimizer = AdamW (Loshchilov and Hutter, 2019)
10. Learning rate = 0.001
11. Weight decay = 0.0001
12. Loss function = BCEWithLogitsLoss with positive-class weighting computed from the training fold

The Transformer encoder was evaluated across seeds 42, 123, 456, 789, and 1,000 with 10-fold stratified cross-validation per seed.

### 2.11 GNNExplainer analysis

Post hoc graph explanations were generated using GNNExplainer through the PyTorch Geometric Explainer interface (Ying et al., 2019). Because the GCN input consisted of integer gene/product identifiers, explanations were generated using learned node embeddings from the trained GCN rather than raw integer node IDs. A wrapper model was used to expose a two-class graph-level output representation for PyTorch Geometric’s explanation interface.

#### 2.11.1 Resistant-genome explanation analysis

For resistant-class interpretation, correctly predicted resistant genomes were selected for explanation. GNNExplainer was run for 200 epochs on graph-level predictions. For each explained graph, node-level importance masks were extracted, converted to CPU tensors, and used to select the top 20 nodes per graph. These nodes were mapped back to Prokka-derived gene/product names using the global vocabulary.

Across explained resistant graphs, node-selection frequency and importance scores were aggregated to generate global GNNExplainer gene/node rankings. The top 50 genes/nodes from this ranking were used for resistance-architecture interpretation, STRING network analysis, Gene Ontology enrichment, downstream computational support, and comparison with Random Forest feature importances. The bulk per-gene GNNExplainer summary and the extended gene-importance statistics from the original resistant-genome explanation workflow are provided in Supplementary Data S1 and Supplementary Data S2, respectively.

#### 2.11.2 Rationale for top-k explanation thresholds

The top-20-per-graph threshold was used to focus on the strongest local explanation signal within each genome graph without allowing very large graphs to dominate the explanation. The top-50 global gene/node ranking was selected to provide enough candidates for network and pathway-level analysis while remaining focused enough for manual biological interpretation. Smaller lists such as top 10 or top 20 may be too restrictive for enrichment analysis, whereas much larger lists may dilute the highest-confidence explanation signal.

#### 2.11.3 Explanation stability across random seeds

Because graph-explanation outputs can vary across models, explainer configurations, and evaluation criteria, explanation stability was quantified rather than inferred from a single trained model (Agarwal et al., 2023). To evaluate explanation stability, five seed-trained GCN models were used. A fixed set of 10 correctly predicted resistant graphs common across all five seed-trained models was selected, with a minimum graph-size threshold of 1,000 nodes to avoid small or incomplete graphs. For each seed-trained model, GNNExplainer was applied to the same selected resistant graphs, producing 50 resistant-class explanation runs.

For each seed, the top 50 explanation genes/nodes were identified. Seed-level top-50 sets were compared using pairwise Jaccard overlap and intersection counts. Consensus sets were computed to identify genes/nodes appearing in the top-50 explanation sets across all five seeds or across at least four or three seeds.

#### 2.11.4 Susceptible-versus-resistant explanation comparison

To address whether explanation patterns were resistant-specific, a parallel susceptible-genome explanation analysis was performed. Ten correctly predicted resistant graphs and ten correctly predicted susceptible graphs that were common across all five seed-trained GCN models were selected. GNNExplainer was then applied across all seed-trained models and both phenotype classes, producing 100 explanation runs. Resistant and susceptible top-50 explanation sets were compared using Jaccard overlap, intersection counts, and class-specific consensus sets.

### 2.12 Random forest-GCN feature comparison

To compare feature-based and graph-based interpretation signals, the top 50 Random Forest features were compared with the top 50 GCN/GNNExplainer-ranked genes/nodes. Random Forest feature importances were derived from the bag-of-genes Random Forest model, whereas GCN/GNNExplainer rankings were derived from aggregated explanation results over correctly predicted resistant graph predictions. For this cross-model comparison, the GCN top-50 set was defined using the frequency-ranked GNNExplainer workflow used for the RF–GCN overlap analysis, while the five-seed explanation-stability consensus ranking was analyzed separately for robustness assessment.

Feature names were normalized where possible to the same Prokka-derived gene/product naming convention. Overlapping features, Random-Forest-specific features, and GCN/GNNExplainer-specific features were then identified. This analysis was used to determine whether the strongest feature-based classifier and the graph-explanation workflow prioritized the same or distinct genomic signals. For functional comparison, each top-50 feature was assigned to Layer 1, Layer 2, Layer 3, or an uncharacterized/putative category based on its Prokka annotation and the same functional definitions used for the three-layer framework; individual assignments are provided in Supplementary Table S11.

### 2.13 Downstream computational analyses

To evaluate whether the GCN/GNNExplainer-prioritized genes formed biologically coherent candidate systems, downstream computational analyses were performed using protein-association enrichment, Gene Ontology enrichment, and molecular docking. These analyses were applied after model training and explanation ranking; they were not used during model fitting or feature selection.

#### 2.13.1 STRING protein-association results

The top 50 GCN/GNNExplainer-prioritized genes/nodes from the resistant-genome explanation analysis were used as input for STRING protein–protein association analysis. Gene/product names were mapped to the closest available *A. baumannii* protein entries where possible. The resulting protein-association network was used to evaluate whether the prioritized genes showed greater functional connectivity than expected by chance. STRING-reported protein–protein interaction enrichment statistics were recorded and used as evidence of network-level coherence among the prioritized genes.

#### 2.13.2 Gene Ontology enrichment

Gene Ontology enrichment analysis was performed on the mapped GCN/GNNExplainer-prioritized genes to identify overrepresented biological processes. Enriched terms were reviewed to determine whether the prioritized genes were associated with functional categories relevant to the proposed resistance-associated architecture, including cell-surface biosynthesis, metabolic regulation, and antibiotic-associated processes. The enrichment analysis was used to evaluate functional organization among the explanation-ranked genes rather than to establish causal biological mechanisms.

#### 2.13.3 Protein-structure preparation

Representative candidate and control proteins were selected for structural analysis based on their roles in the GCN/GNNExplainer-prioritized gene set and their relevance to the proposed resistance-associated layers. AlphaFold2-predicted protein structures were used for docking (Jumper et al., 2021). Representative predicted structures and model-confidence visualizations are shown in Figure 4. Candidate proteins included GDP/UDP-epimerase as a Layer 2 cell-surface biosynthesis candidate and quinone oxidoreductase 1 as a Layer 3 metabolic candidate. OXA-23 was included as a known carbapenem-hydrolyzing class D β-lactamase positive control, using the experimentally determined OXA-23 structure associated with PDB entry 4K0X (Kaitany et al., 2013), and an *A. baumannii* 30S ribosomal protein S12 structure was included as a negative control.

**FIGURE 4:**
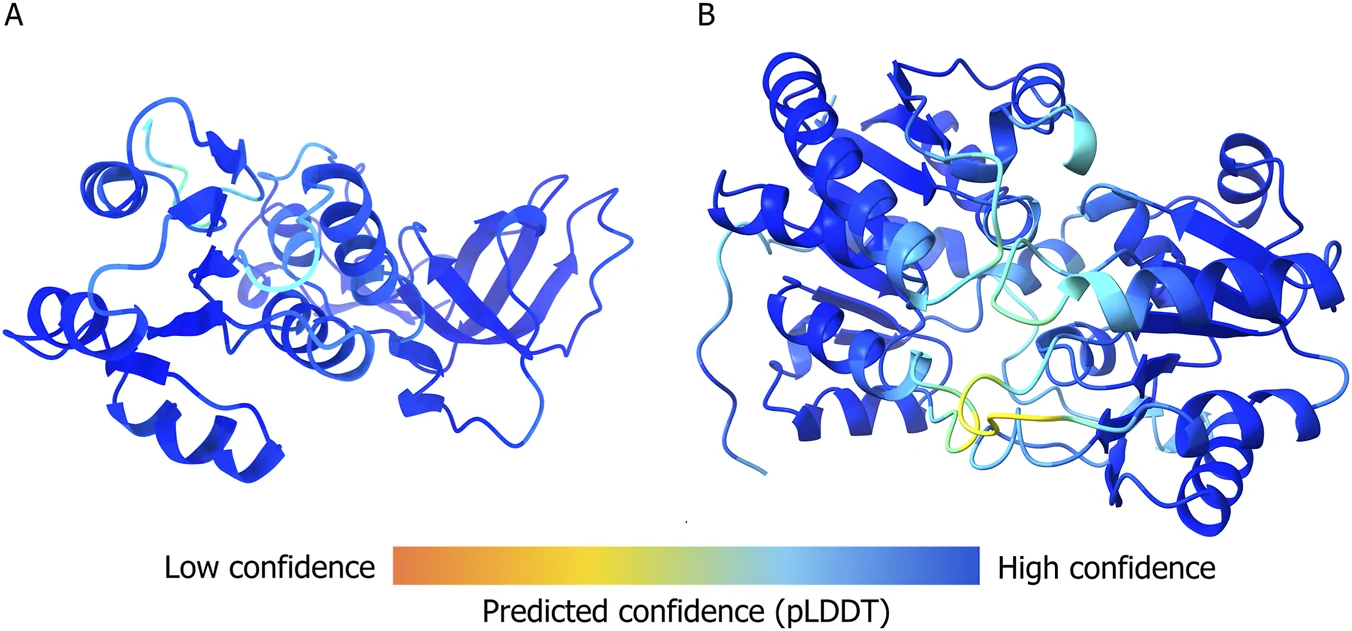
AlphaFold2-predicted structures and model-confidence visualization for GCN-prioritized candidate proteins. **(A)** Rank-1 predicted structure of quinone oxidoreductase 1, a Layer 3 metabolic candidate. **(B)** Rank-1 predicted structure of GDP/UDP-epimerase, a Layer 2 cell-surface biosynthesis candidate. Structures are colored according to predicted Local Distance Difference Test confidence, with blue indicating higher-confidence regions and cyan, yellow, or orange indicating comparatively lower-confidence regions. Both models contain predominantly high-confidence structured regions, with localized lower-confidence segments, and were used for the exploratory molecular-docking analyses.

#### 2.13.4 Molecular docking

Molecular docking was performed using CB-Dock, a cavity-detection-guided blind-docking tool (Liu et al., 2020). Imipenem was selected as a representative carbapenem ligand because the prediction task focused on carbapenem resistance and imipenem was among the carbapenems included during phenotype curation. This choice was also biologically relevant to the structural comparison because OXA-23, the positive-control carbapenemase used here, has experimentally demonstrated hydrolytic activity against imipenem (Kaitany et al., 2013). The carbapenem ligand was docked against each representative candidate or control protein structure. Docking scores were recorded in kcal/mol and compared across candidate, positive-control, and negative-control proteins. The docking analysis was used only to assess structural binding plausibility between carbapenem-like ligands and selected proteins. Docking results were not used to infer biochemical inhibition, causal resistance mechanisms, therapeutic efficacy, or clinical actionability.

### 2.14 Software and reproducibility

Analyses were implemented in Python using custom scripts and were executed primarily in Google Colab, with GPU acceleration where applicable. Graph neural networks and GNNExplainer analyses were implemented using PyTorch and PyTorch Geometric (Fey and Lenssen, 2019), classical machine-learning models were implemented using scikit-learn (Pedregosa et al., 2011), and XGBoost models were implemented using the XGBoost Python package (Chen and Guestrin, 2016). Graph construction, sequence and annotation processing, statistical analysis, and visualization additionally used NetworkX, Biopython, pandas, NumPy, SciPy, and Matplotlib. Available execution metadata verified Python 3.12.13, NumPy 2.0.2, pandas 2.2.2, SciPy 1.16.3, scikit-learn 1.6.1, PyTorch Geometric 2.8.0, and Biopython 1.87 for analyses with retained environment records. Exact versions of PyTorch, XGBoost, NetworkX, and Matplotlib were not retained in the saved execution records and are therefore not reported.

Random seeds were fixed for Python, NumPy, PyTorch, and CUDA where applicable. All reported performance metrics were computed from held-out predictions using the cross-validation and external-validation procedures described above.

Generative AI-assisted code review and debugging were used in support of the computational workflow. All final analyses, outputs, and reported results were reviewed and verified by the authors against the underlying data and retained execution records.

## 3 Results

### 3.1 Overview

Our study produced five major findings. First, model performance was driven primarily by how genome information was represented, with gene-level encodings outperforming k-mer and hybrid schemes. Supervised bag-of-genes models, particularly Random Forest and XGBoost, achieved the strongest internal predictive performance, while the gene-based GCN remained competitive and provided a graph-structured representation for downstream explainability. Second, explainability applied to the graph model prioritized genes consistent with a candidate three-layer resistance architecture spanning canonical resistance, surface biosynthesis, and metabolic regulation. Third, complementary computational analyses, including protein–protein interaction enrichment, GO functional enrichment, and molecular docking, provided computational support for the coherence and structural plausibility of the proposed layers. Fourth, GCN robustness and hyperparameter sensitivity analyses supported the selected GCN training configuration, with performance remaining broadly stable across repeated random seeds and hidden dimensions, while very low learning rate reduced performance. Fifth, external validation on an independent BV-BRC Asia cohort showed reduced generalization for several models, indicating dataset shift between the internal and external cohorts and highlighting the importance of cautious interpretation. Together, these results support a framework in which supervised gene-based classifiers provide strong resistance prediction, while graph-based explainability generates testable systems-level hypotheses.

### 3.2 Comparative performance and the primacy of representation

Figure 3 summarizes the seed-42 internal accuracy comparison across 13 model configurations evaluated using 10-fold stratified cross-validation on the final binary cohort of 2,448 genomes. In this comparison, Random Forest achieved the highest mean accuracy at 0.9359, followed by XGBoost at 0.9261, Logistic Regression at 0.9180, Linear SVM at 0.9081, and the gene-based GCN at 0.8995. Across the original representation benchmark, gene-level representations consistently outperformed k-mer and hybrid graph encodings (Figure 3).

Table 1 presents the final detailed internal benchmark across five random seeds and 10 stratified folds per seed. Random Forest remained the strongest internal classifier, achieving accuracy 0.9328 ± 0.0175, balanced accuracy 0.8988 ± 0.0273, ROC-AUC 0.9650 ± 0.0138, precision 0.9052 ± 0.0413, recall/sensitivity 0.8284 ± 0.0525, specificity 0.9691 ± 0.0149, and resistant-class F1-score 0.8639 ± 0.0367. The standard deviation among the five seed-specific mean accuracies was 0.0026, indicating limited seed-to-seed variation. Figure 3 separately summarizes the seed-42 accuracy-only comparison across all 13 internal model configurations.

The Graph Convolutional Network (GCN), trained on gene-adjacency graphs and evaluated against BV-BRC carbapenem phenotypes classified as resistant or susceptible, also performed strongly. In the repeated-seed robustness analysis, the final gene-based GCN achieved accuracy 0.8963 ± 0.0190, ROC-AUC 0.9460 ± 0.0196, recall/sensitivity 0.7567 ± 0.1024, specificity 0.9450 ± 0.0373, and F1-score 0.7884 ± 0.0460 across five random seeds and 10 stratified folds per seed. These results confirm that gene-level organization contains discriminative structure, even though the strongest predictive performance was achieved by supervised bag-of-genes classifiers.

Logistic Regression provided a strong linear baseline, achieving accuracy 0.9175 ± 0.0206, balanced accuracy 0.8812 ± 0.0311, ROC-AUC 0.9541 ± 0.0187, precision 0.8679 ± 0.0496, recall/sensitivity 0.8061 ± 0.0598, specificity 0.9564 ± 0.0188, and F1-score 0.8343 ± 0.0429 across five random seeds and 10 stratified folds per seed. The standard deviation among the five seed-specific mean accuracies was 0.0027, indicating limited seed-to-seed variation. XGBoost performed similarly to Random Forest, achieving accuracy 0.9297 ± 0.0199, ROC-AUC 0.9646 ± 0.0154, recall/sensitivity 0.8813 ± 0.0516, specificity 0.9467 ± 0.0211, and F1-score 0.8664 ± 0.0376. Linear SVM achieved high specificity but lower sensitivity, indicating a more conservative resistant-class prediction profile.

The exploratory bag-of-genes Transformer encoder achieved accuracy 0.8823 ± 0.0260, ROC-AUC 0.9380 ± 0.0182, and F1-score 0.7813 ± 0.0388. This model used curated Prokka bag-of-genes features tokenized into feature chunks and should therefore be interpreted as a Transformer-style classifier on gene-presence features, not as a raw whole-genome sequence Transformer. The exploratory Random Walk visit-frequency baseline performed weakly, with accuracy 0.5375 ± 0.0337, ROC-AUC 0.5581 ± 0.0410, and F1-score 0.3774 ± 0.0401, indicating that simple random-walk visitation patterns alone did not capture sufficient discriminative signal.

Together, these results show that representation choice was a principal determinant of predictive accuracy, with gene-level encodings capturing the most relevant biological signal. Supervised bag-of-genes models, especially RF and XGBoost, provided the strongest classification performance, whereas the gene-based GCN provided a complementary graph-structured representation for downstream explainability and biological hypothesis generation (Table 1). Model-specific confusion matrices and ROC curves for the gene-based GCN and Random Forest models are provided in Supplementary Figures S1–S4.

### 3.3 External validation on an independent BV-BRC Asia cohort

To assess transfer beyond the internal cohort, the four bag-of-genes classifiers were evaluated on an independent BV-BRC cohort of 2,500 *A. baumannii* genomes assigned to Asia, exceeding the 2,448-genome internal cohort. Following FASTA quality control and Prokka reannotation, the external cohort contained 1,352 susceptible and 1,148 resistant genomes. Of the 5,606 features in the internal Prokka-derived vocabulary, 4,445 were observed at least once in the external cohort, corresponding to 79.29% feature overlap. Each external genome contained a mean of 1,472.7 matched internal-vocabulary features, and the resulting external feature matrix had a density of 26.27%.

Model-specific classification thresholds were selected exclusively from internal out-of-fold predictions and then applied unchanged to the external cohort. Random Forest showed the strongest external performance, achieving an accuracy of 0.7728, balanced accuracy of 0.7765, ROC-AUC of 0.8392, recall/sensitivity of 0.8214, specificity of 0.7315, and F1-score of 0.7685. External ROC-AUC was 0.7666 for XGBoost, 0.7276 for Logistic Regression, and 0.5664 for Linear SVM. At their internally selected thresholds, Logistic Regression, Linear SVM, and XGBoost retained high specificity values of 0.9157, 0.9120, and 0.9164, respectively, but showed substantially lower sensitivities of 0.1516, 0.0270, and 0.1803.

External performance was lower than internal cross-validation performance for all four evaluated models, demonstrating reduced transferability across cohorts. Nevertheless, Random Forest retained the strongest overall discrimination and threshold-based classification performance in the external analysis. Complete external metrics and confusion-matrix counts are reported in Table 2 and Supplementary Table S6, the internally selected thresholds are provided in Supplementary Table S7, and the external ROC curves and Random Forest confusion matrix are shown in Supplementary Figure S5.

**TABLE 2:** External validation performance on the independent BV-BRC Asia cohort.

Model	Threshold	Accuracy	Balanced accuracy	ROC-AUC	Recall/Sensitivity	Specificity	F1
Logistic regression	0.422256	0.5648	0.5336	0.7276	0.1516	0.9157	0.2423
Random forest	0.319965	0.7728	0.7765	0.8392	0.8214	0.7315	0.7685
Linear SVM	−0.331230	0.5056	0.4695	0.5664	0.0270	0.9120	0.0478
XGBoost	0.377530	0.5784	0.5484	0.7666	0.1803	0.9164	0.2820

Models were trained on the internal cohort of 2,448 genomes. Model-specific thresholds were selected exclusively from internal out-of-fold prediction scores by maximizing balanced accuracy and were then applied unchanged to the external cohort of 2,500 genomes. The external cohort contained 1,352 susceptible and 1,148 resistant genomes, with resistance treated as the positive class. Complete precision values and confusion-matrix counts are provided in Supplementary Table S6, and the internally selected thresholds are documented in Supplementary Table S7.

### 3.4 GCN hyperparameter sensitivity and explanation analyses

#### 3.4.1 GCN hyperparameter sensitivity

To assess whether the selected GCN configuration was sensitive to key training choices, we performed a focused analysis across learning rate and hidden-channel dimensionality. At 128 hidden channels, learning rates of 0.01, 0.001, and 0.0001 were compared; at a fixed learning rate of 0.001, hidden sizes of 64, 128, and 256 were compared. The selected configuration of learning rate 0.001, hidden size 128, and 20 training epochs achieved an accuracy of 0.8995 ± 0.0240, balanced accuracy of 0.8541 ± 0.0366, ROC-AUC of 0.9459 ± 0.0172, recall/sensitivity of 0.7598 ± 0.0987, specificity of 0.9483 ± 0.0463, and F1-score of 0.7957 ± 0.0461. Performance was broadly similar across hidden sizes of 64, 128, and 256. Among these hidden-size variants, the selected 128-channel configuration produced the highest balanced accuracy and resistant-class recall, although the 64- and 256-channel configurations showed marginally higher raw accuracy. In contrast, the lowest learning rate tested, 0.0001, substantially reduced recall/sensitivity to 0.4399 ± 0.2095 and F1-score to 0.5543 ± 0.2266. The selected configuration was therefore retained for the repeated-seed benchmark and downstream explanation analyses (Table 3; Figure 5). Complete sensitivity and seed-specific GCN results are provided in Supplementary Table S8.

**TABLE 3:** Focused GCN hyperparameter-sensitivity analysis.

Configuration	Learning rate	Hidden size	Accuracy	Balanced accuracy	ROC-AUC	Recall/ Sensitivity	Specificity	F1
LR high	0.01	128	0.8979 ± 0.0250	0.8499 ± 0.0483	0.9420 ± 0.0172	0.7503 ± 0.1212	0.9494 ± 0.0435	0.7891 ± 0.0582
**Selected GCN**	**0.001**	**128**	**0.8995 ± 0.0240**	**0.8541 ± 0.0366**	**0.9459 ± 0.0172**	**0.7598 ± 0.0987**	**0.9483 ± 0.0463**	**0.7957 ± 0.0461**
LR low	0.0001	128	0.8402 ± 0.0424	0.7097 ± 0.0957	0.9007 ± 0.0245	0.4399 ± 0.2095	0.9796 ± 0.0277	0.5543 ± 0.2266
Hidden small	0.001	64	0.9024 ± 0.0259	0.8513 ± 0.0418	0.9449 ± 0.0174	0.7455 ± 0.1074	0.9570 ± 0.0465	0.7966 ± 0.0538
Hidden large	0.001	256	0.9020 ± 0.0203	0.8485 ± 0.0281	0.9460 ± 0.0169	0.7377 ± 0.0813	0.9592 ± 0.0420	0.7955 ± 0.0376

Learning-rate settings were compared with hidden size fixed at 128, and hidden-channel settings were compared with learning rate fixed at 0.001. Values are reported as mean ± standard deviation across 10-fold stratified cross-validation. All configurations used an embedding dimension of 128, batch size of 16, Adam optimization, BCEWithLogitsLoss, and 20 training epochs. The selected configuration used learning rate 0.001 and hidden size 128. The primary repeated five-seed GCN benchmark is reported in Table 1, and complete sensitivity results are provided in Supplementary Table S8. Resistance was treated as the positive class. Bold values indicate the selected GCN configuration (learning rate = 0.001; hidden size = 128) used for the repeated-seed benchmark and downstream explanation analyses.

**FIGURE 5:**
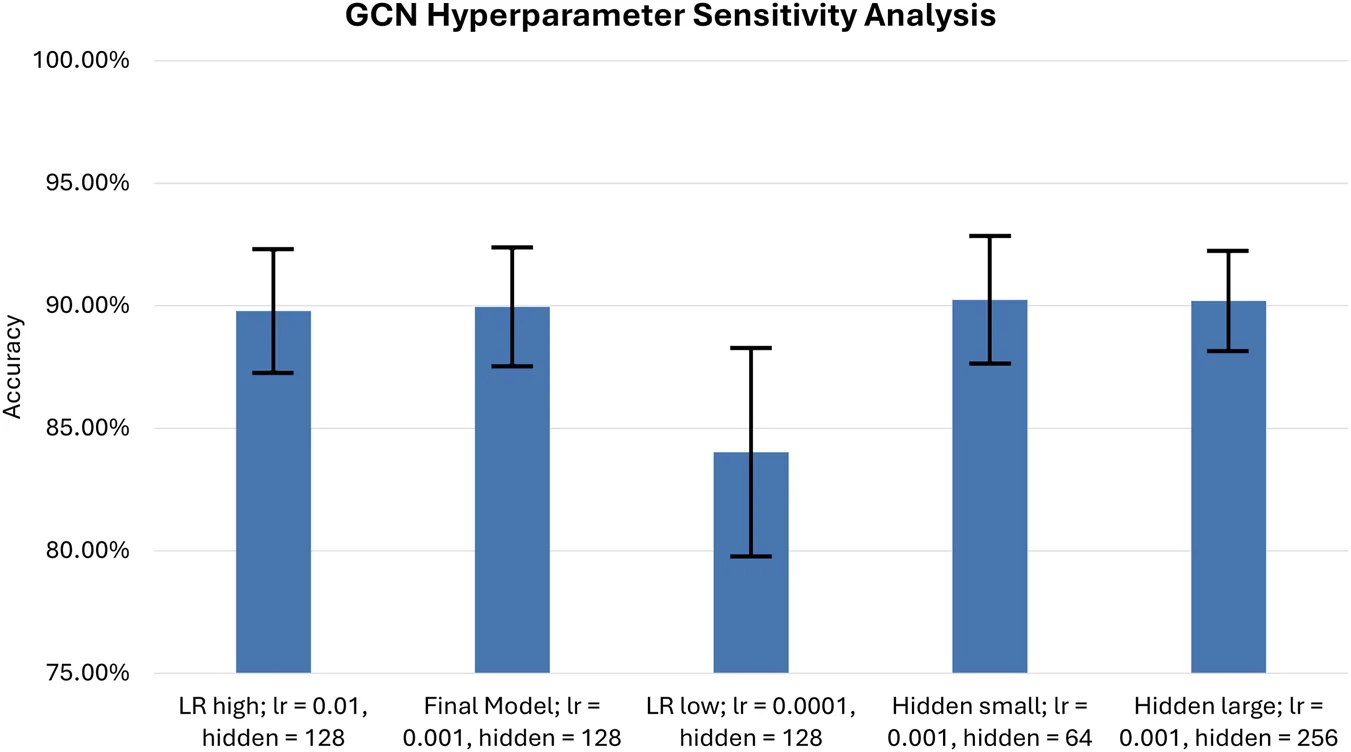
GCN hyperparameter sensitivity analysis. Mean held-out accuracy is shown for five gene-based Graph Convolutional Network configurations evaluated using stratified 10-fold cross-validation on the final binary cohort of 2,448 *Acinetobacter baumannii* genomes. Learning rates of 0.01, 0.001, and 0.0001 were compared with the hidden dimension fixed at 128, while hidden dimensions of 64, 128, and 256 were compared with the learning rate fixed at 0.001. Bars represent mean accuracy across the 10 held-out folds, and error bars represent standard deviation across folds. Performance was broadly stable across learning rates of 0.01 and 0.001 and hidden dimensions of 64, 128, and 256, whereas the learning rate of 0.0001 produced lower and more variable accuracy.

#### 3.4.2 GNNExplainer stability across random seeds

Because GNNExplainer is a stochastic *post hoc* explanation method, we next evaluated whether the explanation patterns were stable across independently seeded GCN models. We applied GNNExplainer to a fixed set of 10 correctly predicted resistant graphs that were common across five seed-trained GCN models. For each graph, the top 20 explanatory nodes were retained, and seed-level top-50 gene/node sets were compared. All 50 explanation runs completed successfully, with no failed explanations. The mean pairwise top-50 Jaccard overlap across seed-trained models was 0.3198 ± 0.0292, with a mean intersection size of 24.20 ± 1.69 genes/nodes. In addition, 19 genes/nodes appeared in the top-50 explanation sets for all five seeds, 24 appeared in at least four seeds, and 28 appeared in at least three seeds. These results indicate that the GNNExplainer outputs contained a reproducible consensus subset, while also showing moderate seed-dependent variability. Accordingly, we interpret GNNExplainer-ranked genes as model-explanation patterns that support hypothesis generation, not as validated causal biomarkers. Seed-level performance, pairwise top-50 Jaccard overlaps, intersection counts, and consensus explanation genes/nodes are provided in Supplementary Table S9. Predictive and explanation stability across the five GCN seeds is visualized in Supplementary Figure S6.

We also performed a parallel GNNExplainer analysis for correctly predicted susceptible genomes to determine whether the graph explanations were resistant-specific or reflected general graph-level prediction features. Using 10 correctly predicted resistant graphs and 10 correctly predicted susceptible graphs common across the five seed-trained GCN models, we generated 100 successful GNNExplainer outputs with no failures. The resistant and susceptible consensus top-50 explanation sets were distinct: the seed-level resistant-vs-susceptible top-50 Jaccard overlap was 0.0 ± 0.0, the mean intersection count was 0.0, and the consensus top-50 comparison contained 50 resistant-specific and 50 susceptible-specific genes/nodes with no shared top-50 genes/nodes. This result suggests that the GCN relied on class-specific explanation patterns for resistant and susceptible predictions, although these patterns should still be interpreted as model-derived signals rather than experimentally validated mechanisms. The resistant-versus-susceptible summary, seed-level overlap results, consensus-set comparison, and class-specific consensus genes/nodes are provided in Supplementary Table S10.

Finally, we compared the top-ranked Random Forest features with the top-ranked GCN/GNNExplainer genes to assess whether the feature-based and graph-based models prioritized the same biological signals. The RF top-50 and GCN/GNNExplainer top-50 sets overlapped by 11 features, leaving 39 RF-specific and 39 GCN-specific entries. Functional categorization of the RF top-50 features identified 16 Layer 1 features (32%), 10 Layer 2 features (20%), 22 Layer 3 features (44%), and two uncharacterized/putative features (4%) (Figure 6B). This partial overlap indicates that the RF and GCN prioritized related but non-identical signals, with the RF emphasizing individual bag-of-genes predictors and the GCN/GNNExplainer analysis highlighting graph-contextual features. The complete Random Forest and GCN/GNNExplainer top-50 ranked lists, individual layer assignments, shared features, model-specific features, and overlap summary are provided in Supplementary Table S11.

**FIGURE 6:**
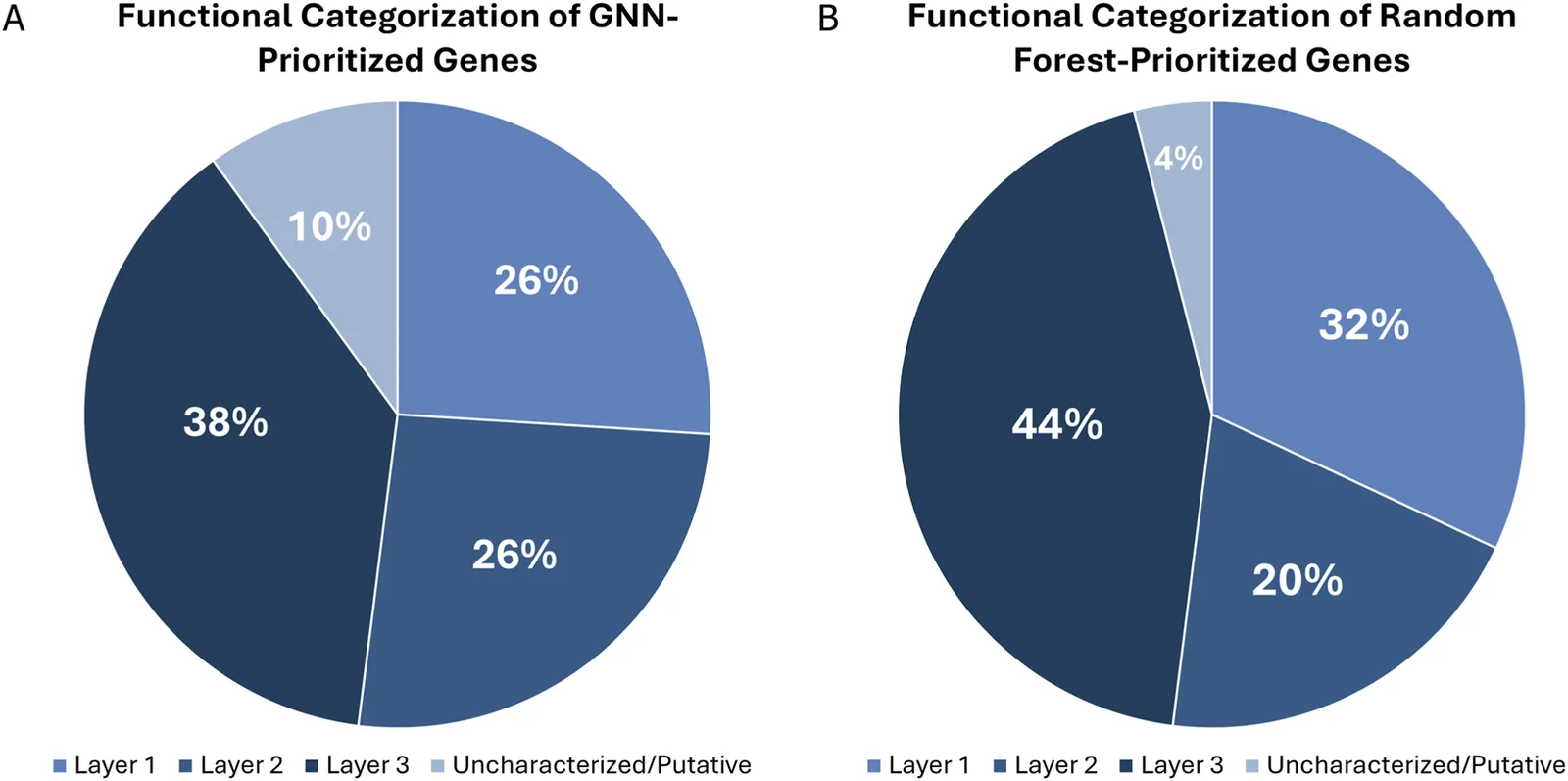
Functional categorization of top-ranked GNNExplainer and Random Forest features. **(A)** The top 50 GNNExplainer-prioritized genes/nodes were assigned to Layer 1 canonical resistance functions, Layer 2 structural and cell-surface functions, Layer 3 metabolic and regulatory functions, or an uncharacterized/putative category. Layer 1 contained 13 of 50 features (26%), Layer 2 contained 13 of 50 (26%), Layer 3 contained 19 of 50 (38%), and five of 50 (10%) were uncharacterized or putative. **(B)** The top 50 Random Forest features were categorized using the same framework. Layer 1 contained 16 of 50 features (32%), Layer 2 contained 10 of 50 (20%), Layer 3 contained 22 of 50 (44%), and two of 50 (4%) were uncharacterized or putative. Individual feature assignments are provided in Supplementary Table S11.

### 3.5 Candidate three-layer resistance architecture from GCN explanations

Although the RF model achieved the strongest predictive performance, the GCN was retained as a complementary graph-based model for mechanism-oriented hypothesis generation. Because the GCN represents genomes as gene-adjacency graphs, GNNExplainer was used to identify model-explanation patterns associated with resistant predictions. These explanations highlighted a candidate three-layer resistance architecture among the top 50 ranked genes/nodes (Figure 7):

- Layer 1 - Canonical Resistance (26%)

**FIGURE 7:**
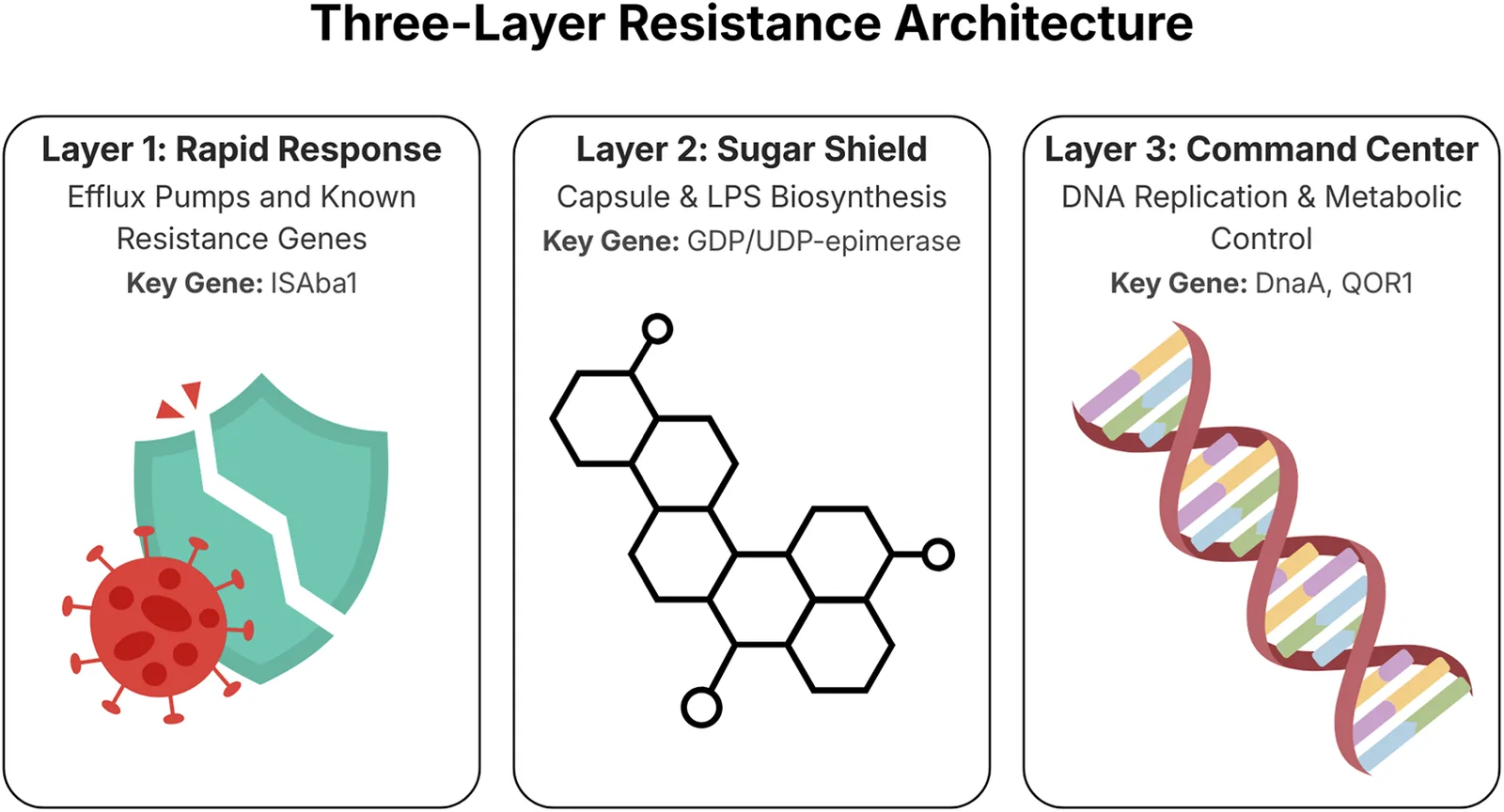
Conceptual model of the Three-Layer Resistance Architecture identified from feature-based and graph-based analyses. Layer 1, termed Rapid Response, includes canonical resistance-associated features such as efflux systems, β-lactamases, and mobile genetic elements, represented by ISAba1. Layer 2, termed the Sugar Shield, includes capsule and lipopolysaccharide biosynthesis features, with GDP/UDP-epimerase highlighted as a representative candidate. Layer 3, termed the Command Center, includes DNA-replication and metabolic-regulation features, represented by DnaA and quinone oxidoreductase 1. Diagram layout, text, and geometric elements were created by the author. Incorporated icons were used under the Canva Pro Content License through Canva for Education and remain subject to Canva’s applicable license terms.

Genes involved in drug modification and mobilization, including the IS4-family transposase ISAba1 (Bartal et al., 2022; Hamidian and Nigro, 2019). These are consistent with an immediate-response defense layer.

- Layer 2 - Structural Reinforcement (26%)

Cell-surface and capsule biosynthetic enzymes, with GDP/UDP-epimerase emerging as a recurrent hub. These genes are consistent with envelope-remodeling functions that may influence antibiotic entry or cell-surface stress responses.

- Layer 3 - Metabolic Rewiring (38%)

Global regulators such as DnaA and quinone oxidoreductase 1 (QOR1), consistent with possible adaptive modulation of replication and energy metabolism during carbapenem stress. Together, Layers 1–3 accounted for 90% of the top 50 GNNExplainer-prioritized genes/nodes, while the remaining 10% were uncharacterized or putative proteins, highlighting a tractable frontier for future mechanistic investigation (Figure 6A).

### 3.6 Computational support for candidate resistance-associated genes

#### 3.6.1 Network coherence (STRING PPI analysis)

STRING analysis mapped 38 of the top 50 GNN-prioritized genes and revealed a significantly enriched protein-association network (PPI enrichment p = 0.00158), indicating greater functional association among the mapped proteins than expected by chance (Figure 8; Szklarczyk et al., 2023).

**FIGURE 8:**
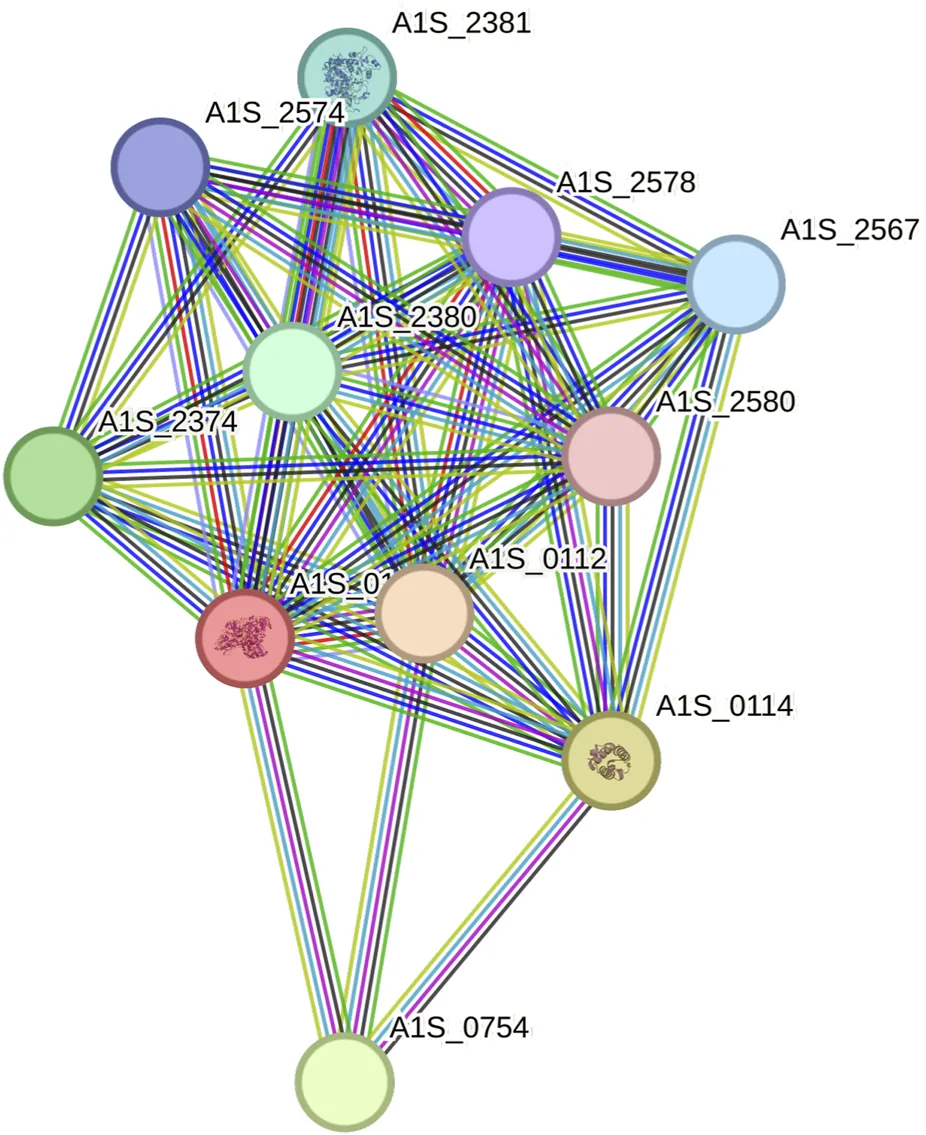
STRING protein-association network for GNNExplainer-prioritized genes. The figure displays the connected component of the mapped STRING network; mapped proteins outside this connected component are not shown. In the full STRING analysis, 38 of the top 50 input genes were mapped, and the resulting protein-association network showed significant enrichment (PPI enrichment p = 0.00158). Nodes are labeled using STRING A1S locus identifiers. Edges represent known or predicted functional associations, with edge colors corresponding to distinct STRING evidence channels; multiple colors between a pair of nodes indicate support from multiple evidence sources. The enrichment indicates that the mapped proteins were more functionally associated than expected by chance.

Gene Ontology enrichment indicated strong overrepresentation of biosynthetic and metabolic processes, including siderophore biosynthesis and antibiotic biosynthesis, consistent with the Layer 2 and Layer 3 functions (Figure 9; The Gene Ontology Consortium, 2019).

**FIGURE 9:**
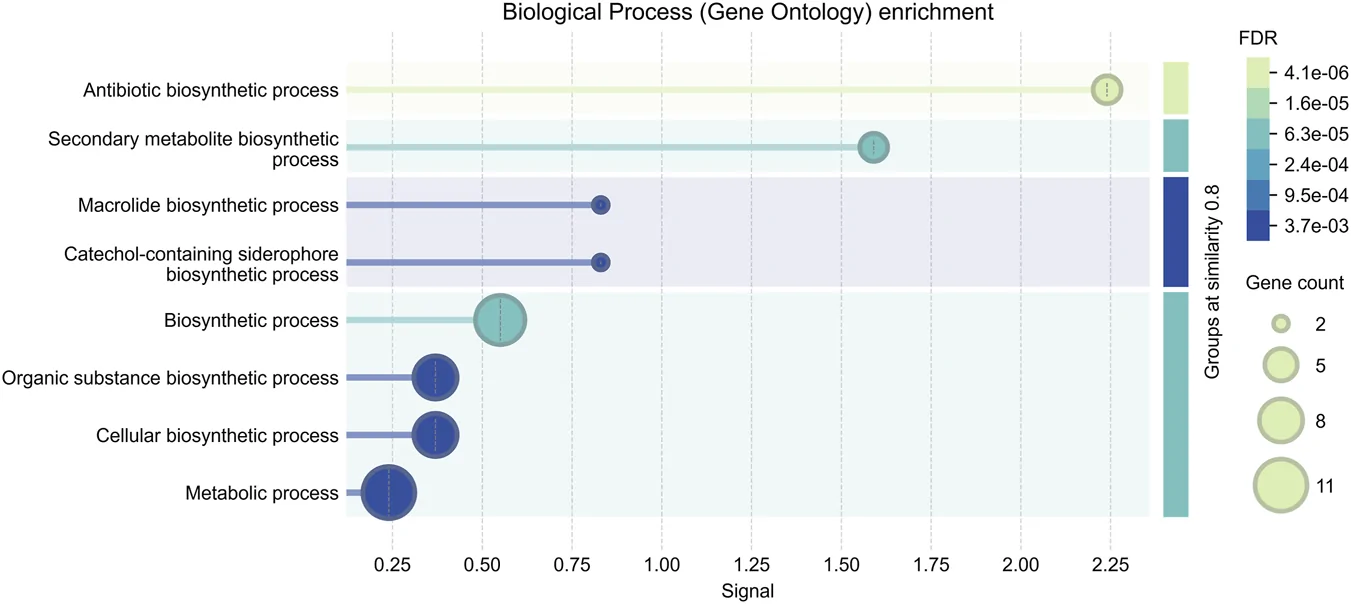
Gene Ontology biological-process enrichment among GNNExplainer-prioritized genes. Enriched biological-process terms are displayed on the vertical axis, and enrichment signal is shown on the horizontal axis. Circle size represents the number of genes associated with each term, while circle color represents the false-discovery rate. Related terms were grouped at a semantic-similarity threshold of 0.8. Enriched categories included antibiotic, secondary-metabolite, macrolide, and catechol-containing siderophore biosynthetic processes, together with broader biosynthetic, cellular, organic-substance, and metabolic processes.

#### 3.6.2 Docking plausibility (structural analysis)

To assess structural plausibility, we docked carbapenem against representative candidates.

- GDP/UDP-epimerase (Layer 2): favorable docking score (−7.2 kcal/mol)
- OXA-23 (Positive control): favorable docking score (−6.7 kcal/mol)
- QOR1 (Layer 3): favorable docking score (−6.6 kcal/mol)
- Negative control (ribosomal protein): less favorable docking score (−5.1 kcal/mol) (Figures 10A–D)

**FIGURE 10:**
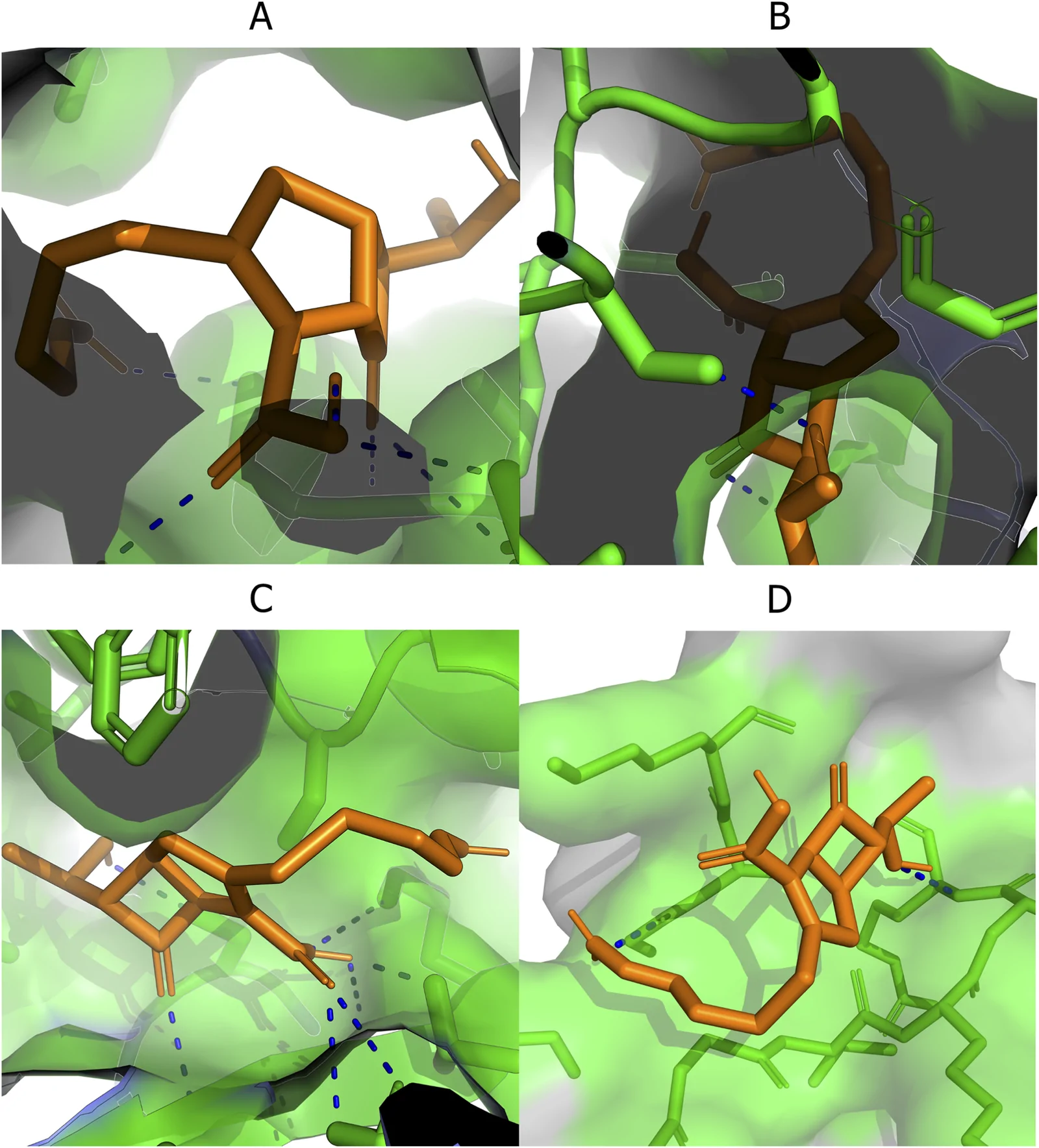
Molecular docking poses of imipenem with GCN-prioritized candidate proteins and positive- and negative-control proteins. **(A)** Imipenem docked within the predicted binding cavity of GDP/UDP-epimerase, with a docking score of −7.2 kcal/mol. **(B)** Imipenem docked within the predicted binding cavity of quinone oxidoreductase 1, with a docking score of −6.6 kcal/mol. **(C)** Positive-control docking of imipenem with the OXA-23 carbapenemase structure (PDB: 4K0X), with a docking score of −6.7 kcal/mol. **(D)** Negative-control docking of imipenem with the *A. baumannii*30S ribosomal protein S12, with a docking score of −5.1 kcal/mol. Imipenem is shown as orange sticks, nearby protein residues as green sticks, the predicted binding cavity as a semitransparent surface, and putative hydrogen-bond interactions as blue dashed lines.

These docking scores are consistent with physically plausible ligand–protein interactions and suggest that the Layer 2 and Layer 3 candidates can accommodate carbapenem-like ligands. However, docking alone cannot establish functional inhibition or biological relevance, and no biochemical activity is claimed.

Together, these findings support a candidate three-layer defense framework in *A. baumannii* integrating established resistance-associated functions with explanation-prioritized structural and metabolic signals for future experimental evaluation.

## 4 Discussion

### 4.1 The prediction-interpretation trade-off

This study addresses two complementary goals in antimicrobial genomics: accurate prediction and biological interpretation. The expanded benchmark showed that supervised Prokka bag-of-genes models, especially Random Forest and XGBoost, achieved the strongest internal classification performance, while the gene-based GCN provided a complementary graph-structured representation for downstream explanation. External validation on an independent BV-BRC Asia cohort further showed that internal cross-validation performance did not fully transfer across cohorts, with Random Forest generalizing best among the externally evaluated models. This pattern highlights both the promise and the limitations of genome-derived resistance prediction: strong internal performance can identify useful predictive signal, but external validation is necessary to evaluate robustness under geographic, annotation, and population-structure differences (Verschuuren et al., 2022; Yu et al., 2025).

Together, the models provide complementary evidence. Feature-based classifiers quantify predictive associations from gene-presence patterns, whereas the gene-adjacency GCN provides a relational representation that can be interrogated using *post hoc* explanation. The GCN should therefore be interpreted as complementary to, rather than a replacement for, the strongest feature-based predictors. Neither model class is inherently mechanistic, and explanation-derived candidates should be treated as hypotheses requiring experimental validation (Agarwal et al., 2023).

### 4.2 Biological interpretation of the candidate three-layer resistance architecture

The GCN explanation results are consistent with a candidate Three-Layer Resistance Architecture in which carbapenem resistance-associated signals may be organized as a coordinated, hierarchical system rather than a loose collection of isolated genes.

- Layer 1 — Canonical defense: Carbapenem-hydrolyzing β-lactamases contribute to drug inactivation, while insertion sequences, transposases, and other mobile genetic elements support the expression, acquisition, and dissemination of resistance determinants. Altered efflux regulation can provide an additional component of the resistance phenotype (Hamidian and Nigro, 2019; Marchand et al., 2004).
- Layer 2 — Structural reinforcement: The explanation analysis prioritized cell-surface and polysaccharide-biosynthesis features, including GDP/UDP-epimerase. Capsule and envelope-biosynthesis pathways have documented roles in stress tolerance, antibiotic susceptibility, and *A. baumannii* envelope homeostasis, supporting the interpretation of these features as candidate surface-remodeling signals (Geisinger and Isberg, 2015; Geisinger et al., 2018; Singh et al., 2019; Akoolo et al., 2022).
- Layer 3 — Metabolic rewiring: The prioritization of DnaA, quinone oxidoreductase 1, and other regulatory or metabolic features is consistent with broader evidence that bacterial replication control and metabolic state can influence responses to antibiotic stress. In this study, these proteins are interpreted as candidate regulatory and metabolic signals rather than established carbapenem-resistance determinants (Skarstad and Katayama, 2013; Spero et al., 2016; Martínez and Rojo, 2011; Acierno et al., 2025).

Together, these layers are consistent with an adaptive feedback-loop model: Layer 1 provides immediate antibiotic neutralization or mobilization-associated defense, Layer 2 supports cell-surface reinforcement, and Layer 3 reflects metabolic and regulatory adaptation that may help maintain cellular viability during antibiotic stress. This systems-level interpretation provides a plausible explanation for how *A. baumannii* may persist under carbapenem pressure even when resistance is not attributable to a single isolated determinant.

The added robustness analyses strengthen this interpretation. GNNExplainer stability testing across five seed-trained GCN models identified a reproducible consensus subset of explanation genes/nodes, while also showing moderate seed-dependent variability. In addition, the susceptible-versus-resistant explanation comparison showed distinct top-50 consensus explanation sets for the two phenotype classes, suggesting that the graph model relied on class-specific explanation patterns rather than a single generic graph signal. The partial overlap between Random Forest top features and GCN/GNNExplainer-ranked genes further supports the complementary nature of the two approaches: feature-based models captured strong individual gene-presence predictors, whereas the GCN highlighted graph-contextual signals that contributed to the proposed architecture. This cautious interpretation is consistent with broader evaluations showing that *post hoc* GNN explanations can vary in fidelity and reliability across methods, datasets, and model configurations (Agarwal et al., 2023).

Comparable combinations of enzymatic antibiotic inactivation, altered membrane permeability, efflux activity, cell-surface remodeling, and regulatory or metabolic adaptation have been described in other Gram-negative pathogens, including *Pseudomonas aeruginosa* and *Klebsiella pneumoniae* (Pang et al., 2019; Navon-Venezia et al., 2017). These parallels support the broader biological plausibility of multilayer resistance responses. However, they do not establish that the specific Three-Layer Resistance Architecture proposed here is conserved across species. Cross-species application of the same modeling and explanation framework will therefore be necessary to determine whether comparable organizational patterns emerge in other pathogens.

### 4.3 Potential translational implications for diagnostics and therapeutic hypothesis generation

The complementary strengths of the RF and GCN models yield two immediate translational pathways.

1. Genomic resistance prediction: The strong internal performance of the Random Forest model, together with its comparatively stronger external validation performance on the independent BV-BRC Asia cohort, supports the potential utility of Prokka-derived gene-presence profiles for rapid carbapenem-resistance prediction. In a future validated workflow, such models could serve as genomic decision-support tools that help prioritize resistant isolates for follow-up testing or surveillance. However, clinical deployment would require prospective validation across hospitals, sequencing platforms, geographic regions, and independently curated phenotypic labels. Previous external-validation studies of WGS-based susceptibility-prediction tools have similarly shown that performance on independent clinical isolates can differ from development-cohort performance, reinforcing the need for prospective and geographically diverse validation before clinical implementation (Verschuuren et al., 2022).
2. Therapeutic hypothesis generation: The GCN/GNNExplainer analysis prioritized genes involved in cell-surface biosynthesis and metabolic regulation, including GDP/UDP-epimerase and quinone oxidoreductase 1. These candidates extend beyond canonical resistance enzymes and suggest that carbapenem resistance may involve coordinated structural and metabolic support systems. Docking results further showed that representative Layer 2 and Layer 3 candidates could accommodate carbapenem-like ligands with scores comparable to the OXA-23 positive control, supporting their structural plausibility as candidates for future experimental investigation. These results do not establish biochemical inhibition or therapeutic efficacy, but they provide a concrete computational basis for testing whether disrupting surface-biosynthesis or metabolic-adaptation components can sensitize *A. baumannii* to carbapenem stress.

### 4.4 Limitations and future directions

Several limitations should be considered when interpreting these results. First, although the internal benchmark showed strong performance, external validation on the independent BV-BRC Asia cohort demonstrated that model generalization varied substantially across classifiers. Random Forest transferred best among the externally evaluated models, whereas Logistic Regression, Linear SVM, and XGBoost showed reduced external sensitivity despite high specificity. This pattern is consistent with domain shift between the internal and external cohorts, potentially reflecting differences in geographic sampling, strain population structure, genome availability, annotation completeness, phenotype-label distributions, and BV-BRC metadata characteristics. These findings support the value of external validation and indicate that future models should be tested prospectively across additional regions, hospitals, sequencing platforms, and independently curated phenotype datasets. Sampling composition and bacterial population structure can also create optimistic internal estimates when closely related lineages are unevenly represented between training and test sets (Yu et al., 2025).

Second, both the internal and external phenotype labels were derived from public database metadata rather than prospective clinical testing performed specifically for this study. BV-BRC labels provide a valuable large-scale resource for computational AMR modeling, but they may include variability in AST methods, reporting standards, antibiotic panels, and metadata completeness. Therefore, the models should be interpreted as computational predictors trained on curated public resistance labels, not as clinically deployable diagnostic tools. Prospective validation against standardized laboratory AST results will be necessary before any clinical decision-support use. BV-BRC integrates large numbers of public genomes and associated metadata from multiple sources, making it valuable for large-scale computational analysis while also inheriting heterogeneity from the underlying submitted records (Olson et al., 2023).

Third, the graph representation simplifies genome organization by treating local gene adjacency as the primary relationship between genes. This representation captures genomic neighborhood structure but does not fully model operon regulation, plasmid mobility, long-range genomic interactions, gene expression, protein abundance, or environmental antibiotic exposure. Future graph models could incorporate plasmid annotations, operon prediction, regulatory networks, protein–protein interactions, or pangenome co-occurrence relationships to represent additional biological structure.

Fourth, although GNNExplainer provided biologically coherent explanation patterns, *post hoc* graph explainability remains sensitive to model initialization and explainer stochasticity. The seed-stability analysis identified a reproducible consensus subset of explanation genes/nodes, but the moderate top-50 Jaccard overlap indicates that individual explanation rankings should not be interpreted as deterministic biomarkers. Similarly, the susceptible-versus-resistant explanation comparison suggests class-specific model-explanation patterns, but these remain computational signals that require biological validation. More generally, explanation agreement and apparent biological plausibility do not by themselves establish explanation fidelity or causal importance (Agarwal et al., 2023).

Fifth, the Random Walk and Transformer analyses were included as exploratory benchmarks. The Random Walk visit-frequency baseline was intentionally simpler than representation-learning approaches such as DeepWalk and node2vec, which combine sampled walks with embedding objectives (Perozzi et al., 2014; Grover and Leskovec, 2016). Its weak performance suggests that simple graph traversal frequencies alone do not capture enough discriminative information for carbapenem-resistance prediction. The Transformer encoder was also exploratory and was inspired by the self-attention architecture of Vaswani et al. (2017), but it used tokenized Prokka bag-of-genes features rather than raw genome sequence. Therefore, it evaluates a Transformer-style classifier within the curated gene-presence feature space, not the broader question of whether raw sequence Transformers can model bacterial AMR.

Finally, the proposed Three-Layer Resistance Architecture remains a computationally supported biological model. STRING, Gene Ontology, and docking analyses support the coherence and structural plausibility of the GCN-prioritized candidates, but they do not establish causal resistance mechanisms, biochemical inhibition, or therapeutic efficacy. Experimental validation using gene knockout, overexpression, complementation, carbapenem susceptibility assays, and mechanistic studies will be needed to test the functional roles of GDP/UDP-epimerase, quinone oxidoreductase 1, and other prioritized genes.

Future work should therefore focus on prospective external validation, lineage-aware evaluation to reduce potential relatedness leakage, richer graph representations, raw-sequence or hybrid sequence-feature Transformer models, and targeted wet-lab validation of the highest-priority candidate genes. By combining expanded benchmarking, external validation, graph explainability, and downstream computational support, this study provides a methodologically transparent foundation for moving from genomic resistance prediction toward experimentally testable mechanistic hypotheses.

## 5 Conclusion

This work presents an integrated computational framework that unites genomic resistance prediction with mechanism-oriented hypothesis generation in antimicrobial resistance. Using 2,448 *Acinetobacter baumannii* genomes, we benchmarked Prokka-derived bag-of-genes models, graph neural networks, and exploratory Random Walk and Transformer-style baselines for carbapenem-resistance prediction. Random Forest achieved the strongest overall performance in the internal benchmark and generalized best in the independent BV-BRC Asia external validation cohort, supporting the value of Prokka-derived gene-presence profiles for resistance prediction and future evaluation as genomic decision-support tools.

The convergence of model benchmarking, external validation, RF–GCN feature comparison, STRING protein-association enrichment, Gene Ontology enrichment, and docking plausibility identifies GDP/UDP-epimerase and quinone oxidoreductase 1 as candidates for future experimental investigation. More broadly, this study illustrates how explainable graph-based modeling can move from descriptive genomic prediction toward mechanism-oriented hypothesis generation, producing computationally grounded candidates that can be tested through targeted biological experiments.

Finally, the core framework used here, pairing high-performing feature-based predictors with graph-based explanation and downstream computational support, is not specific to *A. baumannii*. The same approach could be adapted to other pathogens and resistance phenotypes whenever genome-scale features and meaningful biological relationships can be represented as graphs. As sequencing becomes more accessible, this framework provides a potential template for moving from resistance prediction toward experimentally testable mechanistic hypotheses in complex microbial systems.

## Data Availability

Publicly available genome and antimicrobial-susceptibility data were analyzed in this study. The data were obtained from the Bacterial and Viral Bioinformatics Resource Center (BV-BRC, formerly PATRIC). Genome identifiers and curated phenotype labels for the 2,450-genome internal cohort are provided in Supplementary Table S1, and the final 2,448-genome binary modeling cohort is provided in Supplementary Table S2. Genome identifiers and curated phenotype labels for the independent 2,500-genome external validation cohort are provided in Supplementary Table S12. The supporting model settings, evaluation results, explanation analyses, and derived supplementary outputs are included in the article and Supplementary Material. The custom analysis scripts and notebooks used for data processing, model evaluation, explanation analysis, and figure generation are available from the corresponding author upon reasonable request. Further inquiries can be directed to the corresponding author.
