# Supplementary material for: A comparative analysis of graph neural networks and classical machine learning for carbapenem-resistance prediction and genomic interpretation in Acinetobacter baumannii
Source: Front Bioinform. 2026 Sep 28;6:1885813. doi: 10.3389/fbinf.2026.1885813 (PMC13655062; doi:10.3389/fbinf.2026.1885813)
Supplement: Supplementary file 1 [file Table13.docx]

**Supplementary Figures**

**Supplementary Figure S1. Confusion matrix for the seed-42 gene-based Graph Convolutional Network.** The confusion matrix summarizes complete out-of-fold predictions from 10-fold stratified cross-validation on the final binary cohort of 2,448 Acinetobacter baumannii genomes, comprising 1,815 susceptible and 633 resistant genomes. Rows indicate true phenotypes and columns indicate predicted phenotypes, with susceptible encoded as 0 and resistant encoded as 1. Predictions were assigned using a probability threshold of 0.5, with resistance treated as the positive class. The model correctly classified 1,706 susceptible genomes and 497 resistant genomes, while 109 susceptible genomes were classified as resistant and 136 resistant genomes were classified as susceptible. Supplementary Figures S1 and S2 were regenerated as a separate complete out-of-fold seed-42 evaluation on the corrected 2,448-genome cohort. The primary repeated five-seed benchmark reported in Table 1 and Supplementary Table S8 was retained from the five-seed robustness workflow and is unchanged.

**Supplementary Figure S2. Receiver operating characteristic curve for the seed-42 gene-based Graph Convolutional Network.** The receiver operating characteristic curve was generated from complete out-of-fold resistance probabilities obtained through 10-fold stratified cross-validation on the final binary cohort of 2,448 Acinetobacter baumannii genomes. Each genome received exactly one held-out prediction, and resistance was treated as the positive class. The complete out-of-fold ROC-AUC was 0.9430. The dashed diagonal line represents chance-level discrimination with an ROC-AUC of 0.50. Supplementary Figures S1 and S2 were regenerated as a separate complete out-of-fold seed-42 evaluation on the corrected 2,448-genome cohort. The primary repeated five-seed benchmark reported in Table 1 and Supplementary Table S8 was retained from the five-seed robustness workflow and is unchanged.

**Supplementary Figure S3. Confusion matrix for the Random Forest classifier.** The confusion matrix summarizes out-of-fold predictions for the final binary cohort of 2,448 *A. baumannii* genomes under 10-fold stratified cross-validation. In this figure, rows indicate true phenotypes and columns indicate predicted phenotypes, ordered as resistant followed by susceptible. The model correctly classified 532 resistant genomes and 1,764 susceptible genomes, while 101 resistant genomes were classified as susceptible and 51 susceptible genomes were classified as resistant.

**Supplementary Figure S4. Receiver operating characteristic curve for the Random Forest classifier.** The receiver operating characteristic curve was generated from pooled out-of-fold prediction scores from the seed-42, 10-fold stratified cross-validation evaluation of the final binary cohort of 2,448 *Acinetobacter baumannii* genomes. The Random Forest classifier achieved an area under the curve of 0.9657. The dashed diagonal line represents chance-level discrimination with an area under the curve of 0.50.

**Supplementary Figure S5. External validation on the independent BV-BRC Asia cohort. (A)** Receiver operating characteristic curves for Linear SVM, Logistic Regression, Random Forest, and XGBoost evaluated on an independent external cohort of 2,500 *Acinetobacter baumannii* genomes from Asia. The models were trained using the internal 2,448-genome cohort, and prediction scores were generated for the external cohort. Random Forest achieved the highest external ROC-AUC of 0.8392, followed by XGBoost at 0.7666, Logistic Regression at 0.7276, and Linear SVM at 0.5664. The dashed diagonal line represents chance-level discrimination. **(B)** Confusion matrix for Random Forest predictions on the external cohort using the classification threshold selected exclusively from internal cross-validation predictions. The selected threshold was 0.319965 and is displayed as 0.320 in the figure. Rows indicate true phenotypes and columns indicate predicted phenotypes. Random Forest correctly classified 989 susceptible and 943 resistant genomes, while 363 susceptible genomes were classified as resistant and 205 resistant genomes were classified as susceptible.

**Supplementary Figure S6. Predictive and explanation stability across GCN random seeds. (A)** Mean accuracy of the gene-based Graph Convolutional Network across random seeds 42, 123, 456, 789, and 1000. Stratified 10-fold cross-validation was conducted independently for each seed on the final binary cohort of 2,448 *A. baumannii* genomes. Bars represent mean held-out accuracy across the 10 folds, and error bars represent the corresponding standard deviation. **(B)** Pairwise Jaccard overlap among the seed-specific top-50 GNNExplainer gene/node sets produced by the five independently trained GCN models. Explanations were generated using the same fixed set of 10 correctly predicted resistant graphs for each seed-trained model. Diagonal values represent self-comparisons, while off-diagonal values quantify overlap between the top-50 sets from different random seeds.

**Supplementary Data**

**Supplementary Data S1. Bulk GNNExplainer gene-importance results.** Per-gene summary of the GNNExplainer outputs, including selection frequency, mean importance, maximum importance, standard deviation of importance, and median importance across explained graphs.

**Supplementary Data S2. Comprehensive GNNExplainer gene-importance analysis.** Extended per-gene explanation summary including frequency and frequency percentage, importance-score statistics, average and extreme rank positions, coefficient of variation, score range, consistency score, and rank-adjusted score.

**Supplementary Tables**

**Supplementary Table S1. Final curated carbapenem phenotype dataset.** List of the 2,450 *Acinetobacter baumannii* genomes retained after carbapenem-phenotype curation. The table includes genome identifiers, genome names, final carbapenem phenotype, inclusion in the curated dataset, binary-modeling status, and reason for exclusion from binary modeling where applicable. Two intermediate-phenotype genomes were retained in the curated dataset but excluded from the binary modeling cohort.

**Supplementary Table S2. Final binary modeling dataset.** List of the 2,448 *A. baumannii* genomes used for model training and evaluation after exclusion of two intermediate-phenotype isolates. The final binary cohort contained 1,815 susceptible and 633 resistant genomes.

**Supplementary Table S3. Dataset, feature-matrix, and gene-graph summary statistics.** Summary of the internal carbapenem-phenotype cohort, Prokka-derived bag-of-genes matrix, binary gene-graph dataset, and independent BV-BRC Asia external-validation cohort. Reported values include phenotype counts, bag-of-genes feature counts, graph node and edge distributions, external cohort size and class composition, and overlap between the external features and the internal 5,606-feature vocabulary.

**Supplementary Table S4. Model architecture, hyperparameter, and evaluation settings.** Implementation details for the complete 13-configuration seed-42 benchmark and the seven principal models included in the repeated five-seed benchmark. The table documents the four bag-of-genes classifiers; GCN, GraphSAGE, and GAT across gene-based, k-mer-based, and hybrid graph representations; the Random Walk visit-frequency baseline; and the bag-of-genes Transformer encoder. It also reports model-specific hyperparameters, optimization settings, class-weight handling, cross-validation design, random seeds, and external-validation configurations where applicable. The table reports model-specific hyperparameters, optimization settings, class-weight handling, cross-validation design, random seeds, and external-validation configurations.

**Supplementary Table S5. Complete internal model benchmark metrics.** The workbook contains two worksheets. (A) Mean held-out accuracy for all 13 internal model configurations evaluated using random seed 42 and stratified 10-fold cross-validation. (B) Complete repeated five-seed metrics for the seven principal models reported in Table 1, including accuracy, ROC-AUC, precision, recall/sensitivity, specificity, F1-score, and aggregate confusion-matrix counts. Values in the five-seed worksheet are reported as mean ± standard deviation across five random seeds and 10 stratified folds per seed.

**Supplementary Table S6. External-validation performance on the independent BV-BRC Asia cohort.** Performance of Logistic Regression, Random Forest, Linear SVM, and XGBoost on the 2,500-genome external cohort, which contained 1,352 susceptible and 1,148 resistant genomes. Models were trained on the internal cohort, and internally selected classification thresholds were applied unchanged to the external data. The table reports threshold, accuracy, balanced accuracy, ROC-AUC, precision, recall/sensitivity, specificity, F1-score, and confusion-matrix counts.

**Supplementary Table S7. Internally selected classification thresholds used for external validation.** Model-specific thresholds were selected exclusively from out-of-fold scores generated by 10-fold stratified cross-validation in the 2,448-genome internal cohort. Candidate thresholds were evaluated by balanced accuracy, and the threshold producing the highest internal balanced accuracy was selected for each model. Predicted resistance probabilities were used for Logistic Regression, Random Forest, and XGBoost, whereas decision-function scores were used for Linear SVM. The selected thresholds were applied unchanged to the independent 2,500-genome external cohort, with resistance treated as the positive class.

**Supplementary Table S8. GCN repeated-seed robustness and hyperparameter sensitivity.** The workbook contains two worksheets. **(A)** Performance of the selected gene-based GCN across random seeds 42, 123, 456, 789, and 1000, with 10-fold stratified cross-validation conducted for each seed. **(B)** Focused sensitivity analysis comparing learning rates of 0.01, 0.001, and 0.0001 and hidden dimensions of 64, 128, and 256. Metrics are reported as mean ± standard deviation.

**Supplementary Table S9. GNNExplainer seed-stability analysis.** The workbook contains the overall explanation-stability summary, pairwise top-50 Jaccard overlaps and intersection counts across five seed-trained GCN models, and consensus genes/nodes appearing across multiple seed-level top-50 explanation sets. The analysis used the same 10 correctly predicted resistant graphs across all five trained models.

**Supplementary Table S10. Resistant-versus-susceptible GNNExplainer comparison.** Comparison of GNNExplainer-ranked genes/nodes from 10 correctly predicted resistant genomes and 10 correctly predicted susceptible genomes common across five seed-trained GCN models. The workbook includes the overall comparison summary, seed-level top-50 overlaps, consensus-set overlap statistics, and phenotype-specific consensus gene/node lists.

**Supplementary Table S11. Comparison of the top 50 Random Forest and GCN/GNNExplainer features.** Ranked top-50 feature lists from the Random Forest and GCN/GNNExplainer analyses, together with normalized feature names, model-specific ranks, individual annotation-based functional-layer assignments under the proposed three-layer framework, shared features, and features uniquely prioritized by each model. The two top-50 sets contained 11 shared features, with 39 features unique to each model. Layer assignments represent annotation-based functional categorizations under the proposed framework.

**Supplementary Table S12. External validation cohort genome identifiers and curated labels.** The table lists the 2,500 BV-BRC *Acinetobacter baumannii* genomes included in the independent external validation cohort, comprising 1,352 susceptible and 1,148 resistant genomes. Binary labels are defined as 0 for susceptible and 1 for resistant.
